# Search and rescue system-of-systems influence degree evaluation of aviation equipment based on simulation

**DOI:** 10.1038/s41598-022-26098-x

**Published:** 2022-12-26

**Authors:** Yan Gao, Hu Liu, Fu Niu, Yongliang Tian, Jin Wang, Wangchi Cheng

**Affiliations:** 1 School of Aeronautic Science and Engineering, Beihang University, Beijing, China; 2 Academy of Systems Engineering, Beijing, China

**Keywords:** Engineering, Mathematics and computing

## Abstract

Search and rescue (SAR) is an important part of joint operations, and also one of the key supports for ensuring combat effectiveness. Aviation equipment is a major component of SAR action. Therefore, the SAR capability of aviation equipment has become the key to affecting the overall SAR action. This paper proposes the concept of the system of systems influence degree (SoSID) and conducts a scientific quantitative evaluation to quantitatively measure the effect of aviation equipment used in SAR. First, according to the characteristics of SAR action in threat environments, the SAR capability of aviation equipment is analyzed, and an indicator decomposition hierarchy model based on this SAR capability is proposed. Second, based on the above model, the DECIDE (destroy, execute, cost, implement, defend, evade) SoSID evaluation model is proposed. Third, a comparative test is designed and a sensitivity analysis is conducted based on the sobol power sensitivity (SPS) analysis method to obtain the index sensitivity of the SAR capability. The sensitivity is then ranked to obtain key indicators. Finally, we build a simulation test environment to obtain multiple test plans for comparison and verify the rationality of the index decomposition hierarchy model and the SoSID evaluation model as well as the effectiveness of the SPS analysis method through analysis of the simulation results.

## Introduction

In modern high-tech wars and local conflicts, the search and rescue (SAR) for people in distress on one’s side not only acts to save an individual life but also often develops into severe military and political events. Therefore, it is of great significance to successfully conduct SAR actions to improve the operation capability of the joint force.

SAR mainly consists of SAR teams, including helicopters, attack aircraft and refueling aircraft, radar satellites and other auxiliary forces, including mission control and rescue coordination. Aviation equipment system SAR teams have been the key force for a SAR action.

Therefore, this paper places aviation equipment into a SAR mission, through simulation test environment and analyzing the simulation data to calculate the influence degree of each piece of aviation equipment and its performance on the SAR action, so as to provide a theoretical reference for the design of aviation equipment and decision support for the commanders faced with actual SAR mission.

A common approach to measuring the degree of the influence of aviation equipment on the system of systems (SoS) state is to introduce the concept of SoS contribution, which examines the contribution degree of changes of the equipment to be evaluated to the effectiveness of the operation SoS. Measurement of the SoS contribution of equipment, based on a review of the current domestic and international studies, is mainly based on the perspectives of the SoS mission effectiveness and the SoS requirement satisfactory degree, which mainly includes the contributions of weapon and equipment to the combat mission accomplishment effect^1,2^, the optimization of combat SoS structure^3–5^, and the benefit of SoS construction^6^, but mature metrics and related theoretical methods have not yet been developed. From the perspective of SoS mission effectiveness, Luo et al.^7^ discussed the equipment evaluation method based on “exploratory analysis+” from four dimensions, namely, task, capability, structure, and evolution. Zhu et al.^8^ and Sun et al.^9^ combined a new type of armor system contribution evaluation task with the advantage of the repeatability of simulation experiments, and carried out equipment SoS contribution evaluation by correlation analysis and cause-effect retrospective analysis. Luo et al.^10^ initially performed an evaluation for weapon and equipment combat SoS contribution by the structural equation modeling (SEM) method to establish a weapon and equipment combat SoS contribution evaluation index system. From the perspective of SoS requirement satisfactory degree, Golany et al.^11^ established a network optimization model to solve the problem of equipment development planning under resource constraints. Zhao et al.^12^ introduced the ideas of the mission and method framework (MMF) and the feedback mechanism, and proposed a value engineering-oriented equipment SoS contribution analysis method according to the characteristics of the equipment SoS and the analytical ideas of complex systems. Wang et al.^13^ and Sun et al.^14^ proposed a research method for the contribution of equipment system function to the SoS, and used a fuzzy index scale to construct an index system, so as to analyze the contribution of system function to the SoS. Lee^15^ proposed a method for assessing the contribution of equipment based on the weapon and equipment SoS combat network model.

There are two characteristics of the above studies regarding the SoS contribution of equipment:

First, these studies mainly measure the contribution degree of equipment to the SoS effectiveness by measuring the change rate of the SoS mission execution level before and after the equipment being evaluated is incorporated into the SoS or by comparing the difference of SoS status with and without the equipment being evaluated.

However, the measurement of the role or influence of the equipment on the SoS reflects the relationship between the equipment and the SoS when oriented to a specific mission. Thus, in terms of SAR, when facing a specific SAR mission with relatively fixed equipment types, it is necessary to formulate the corresponding SAR force allocation (i.e., choose a certain combination of equipment), which involves the consideration of the scale effect of a certain type of equipment, in other words, it is necessary to examine the influence of different equipment quantities (i.e., different SAR force allocations) on the SoS, and is no longer a simple issue of the presence or absence or change of a certain piece of equipment. Therefore, this paper innovatively proposes the concept of the SoSID to comprehensively measure the influence degree of equipment quantity and performance changes on the SoS capability to provide a certain reference value for the equipment users and designers.

Second, these studies measure the SoS contribution through the influence degree of equipment on specific mission effectiveness of SoS or requirement satisfactory degree, which makes it difficult to evaluate the influence degree of equipment on the SoS from multiple levels, dimensions and perspectives.

However, the complexity and multilevel joint characteristics of SAR result in an evaluation of the SAR SoS from the perspective of mission effectiveness or requirement satisfactory degree alone is not comprehensive enough. Meanwhile, there are many factors that affect a SAR operation. It is difficult to obtain the influence of a certain factor on the entire operation through pure mathematical calculation methods or theoretical analysis methods. Simulation methods can be used to study nonlinear and nonmonotonic models^16^, and all input parameters can be changed at the same time, so the model input space is larger, and the analysis results show a better comparison. Therefore, this paper carries out simulation experiments for SAR, and measures the influence degree of aviation equipment and their performance on SAR SoS through simulation so as to provide commanders with strategic support when facing actual SAR missions and provide an important reference for the development of future aviation equipment.

In summary, this paper first proposes the concept of the “SoSID” involving the consideration of the scale effect of a certain type of equipment to comprehensively measure the influence degree of equipment quantity and performance changes on the SoS capability, which provides a certain reference value for the equipment users and the designers. Second, proposes an indicator decomposition hierarchy model based on SAR capability and a DECIDE SoSID evaluation model considering the complex nonlinear relationship between SoS capability and sub-capabilities to multi-dimensionally and multi-levelly analyze the SoSID. Third, proposes the SPS analysis method, which is more suitable for the SOSID evaluation of this kind of SoS with much uncertainty, and not only reduces the number of indicators being subjectively assigned weights and improves the accuracy but also the quasirandom sequence in the sobol sequence sampling method generates random values with uniform distribution that overcomes the drawbacks such as the existence of “gaps” within the sample points brought by pseudorandom sequence sampling. Finally, a case study is conducted through a specific SAR scenario, and the rationality and effectiveness of the proposed model and method are verified .

## Indicator system

### Analysis of SAR capability

Capability analysis is the core link between strategic objectives and development programs for aviation equipment, and is used to clarify the capabilities required for aviation equipment to accomplish mission and to determine indicators reflecting capabilities and the specific equipment and its performance to achieve capabilities^17,18^.

According to reference^19^, the survival rate of injured personnel after 24 h is reduced by 80%, and the survival rate of uninjured personnel after 3 days will decrease significantly. When the rescue time over 5 h, the possibility of survivors being successfully rescued is 20%. If the rescue time is reduced to 1.8 h, the probability of a successful rescue is increased to 60%, which means that the time is reduced by 1/3 and the possibility of distressed personnel being rescued is increased by a factor of 3. Thus, time is the first factor of a SAR mission, and it is crucial to reduce the rescue time to ensure the smooth implementation of SAR action. Second, in the process of a SAR, if there is damage of SAR equipment or casualties of SAR personnel, instead of achieving the purpose of successfully SAR personnel in distress, it will cause greater losses. Third, rescuing as many casualties as possible means that the possibility of personnel being captured is greatly reduced, which is conducive to improving the morale and combat capability of the side.

Therefore, the core of a SAR is a fast, efficient, successful, and safe process, which means using as few SAR forces as possible, in the shortest possible time, to successfully implement SAR for the greatest number of combat casualties.

### Indicator system constructed based on SAR capability

Before analyzing or evaluating the SoSID of aviation equipment, an indicator system needs to be constructed first of all. However, a SAR is a complex activity, and any meaningful analysis or evaluation study is targeted. Therefore, to analyze the SoSID of aviation equipment on a SAR operation, it is necessary to construct an indicator system in a selective manner according to the research objectives, the needs of analysis or evaluation, and the level and content of analysis.

**Figure 1:**
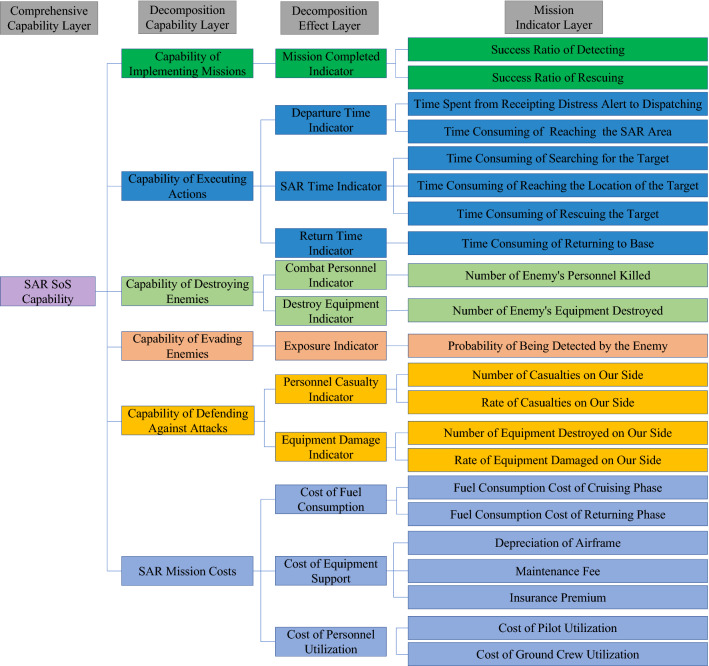
Indicator decomposition hierarchy model.

Through the above analysis, the evaluation of the SoSID of aviation equipment on SAR operation is considered from several aspects, including mission accomplishment, time, and safety. Furthermore, in order to reduce the coupling degree between indicators, the indicators are adjusted, and an indicator decomposition hierarchy model is established with a core of six major capabilities: implementing missions, executing actions, destroying enemies, evading enemies, defending against attacks and SAR mission costs. Specifically, the SAR SoS capability is decomposed into these six sub-capabilities, and then the layers of capabilities are decomposed layer by layer until the specific mission indicators as shown in Fig. 1.

## SoSID evaluation model

Based on the above analysis, this paper first proposes the definition of the SoSID as the influence degree of changes in the quantity (or performance) of the equipment to be evaluated on the SoS capability. We also propose an influence degree calculation model based on the SAR capability, as shown in Eqs. (1) and (2).

1 $$ {\text{inf}}_{{{\text{C}}_{{\text{i}}} }} = \frac{{C_{{{\text{i}} + e_{j} }} - C_{i} }}{{C_{i} }}, $$

2 $$ \inf_{SoS} = \prod {\inf_{{C_{i} }}^{{w_{i} }} } , $$

Equations (1) and (2) are the *i*th sub-capability influence degree calculation equation and SoS capability influence degree calculation equation, respectively. where $\inf_{{C_{i} }}$ is the sub-capability change rate that is the sub-capability influence degree, $C_{i}$ is the value of the *i*th sub-capability without equipment $e_{j}$(or before a performance change of equipment $e_{j}$), $C_{{i + e_{j} }}$ is the value of the *i*th sub-capability after adding equipment $e_{j}$(or after a performance change of equipment $e_{j}$), $\inf_{SoS}$ is the SoS capability change rate that is the SoSID, and $w_{i}$ is the weight value of the sub-capability influence degree $\inf_{{C_{i} }}$.

The SAR SoS capability mainly involves the above six sub-capabilities: destroying enemies, executing actions, SAR mission costs, implementing missions, defending against attacks, and evading enemies, thus the evaluation model can be refined as DECIDE SoSID model, as shown in Eq. (3) and Eq. (4).

3 $$ \inf_{SoS} = f(\inf_{{D_{{{1}_{{}} }} }} ,\inf_{{E_{{1}} }} ,\inf_{C} ,\inf_{I} ,\inf_{{D_{2} }} ,\inf_{{E_{2} }} ), $$

4 $$ \inf_{SoS} = k\inf_{{D_{{1}} }}^{{w_{{D_{1} }} }} \cdot \inf_{{E_{{1}} }}^{{w_{{E_{1} }} }} \cdot \inf_{C}^{{w_{C} }} \cdot \inf_{I}^{{w_{I} }} \cdot \inf_{{D_{{2}} }}^{{w_{{D_{2} }} }} \cdot \inf_{{E_{{2}} }}^{{w_{{E_{2} }} }} , $$

where $\inf_{SoS}$ is the SoSID; $\inf_{{D_{{1}} }}$ is the influence degree of destroying enemies; $\inf_{{E_{{1}} }}$ is the influence degree of executing action; $\inf_{C}$ is the influence degree of SAR mission cost; $\inf_{I}$ is the influence degree of implementing mission; $\inf_{{D_{{2}} }}$ is the influence degree of defending against attacks; $\inf_{{E_{{2}} }}$ is the influence degree of evading enemies; $w_{{D_{{1}} }}$, $w_{{E_{{1}} }}$, $w_{C}$, $w_{I}$, $w_{{D_{{2}} }}$, $w_{{E_{{2}} }}$ are the corresponding weights of $\inf_{{D_{{1}} }}$, $\inf_{{E_{{1}} }}$, $\inf_{C}$, $\inf_{I}$, $\inf_{{D_{{2}} }}$, $\inf_{{E_{{2}} }}$, respectively; and *k* is the correction coefficient.

For ease of description, this paper uses $C_{i}$ (${1} \le i \le {6}$) to represent the capability of implementing mission, the capability of executing action, the capability of destroying enemies, the capability of evading enemies, the capability of defending against attacks, the SAR mission cost, respectively; and $\inf_{{C_{i} }}$ (${1} \le i \le {6}$) is the influence degree value of $C_{i}$.

The traditional SoS contribution is obtained through weighted summation of each specific indicator when calculating the SoS effectiveness, which leads to the subjective assignment of too many specific indicator weights, and increases the error due to subjective factors and will also leads to a decrease in the accuracy of the evaluation model due to different reference criteria when normalizing different types of indicators.

The DECIDE SoSID evaluation model first calculates the influence degree value of the added equipment to the sub-capability, and then conducts weighted synthesis to obtain the change value of the comprehensive capability of the SAR SoS. On one hand, this process requires no normalization and the number of indicators that need to be assigned weights is lower. As seen from Table 1, compared to the traditional SoS contribution calculation method, adopting the DECIDE evaluation model can reduce the number of indicators being assigned weights by 45.5%. On the other hand, according to the aggregation relationship of SAR indicators, the power index method is used for each sub-capability and its influence degree, which can solve the problem that not all indicators within the same indicator layer are adopted during the analysis process. Compared with the calculation method of weighted summation, the DECIDE evaluation model has prominent advantages because of the smaller cumulative error and higher accuracy.

**Table 1:** The difference of the number of indicators being assigned weights between traditional SoS contribution calculation method and SPS analysis method.

Method	Number of indicators being assigned weights
Traditional SoS contribution calculation method	22
SPS analysis method	12

## SPS sensitivity analysis

### SPS analysis method

Sensitivity analysis is an important basis for equipment SoS construction and SoS structure optimization^20,21^. Through sensitivity analysis, the influence degree of weapon and equipment on the SoS can be calculated, and the indicators that have a greater influence on the SoS can be determined, which provides guidance for the construction of the equipment SoS^22^.

Existing sensitivity analysis methods usually fixes the remaining input variables, changes only the value of the single input variable to be studied, and the change of output is the result of sensitivity analysis of that variable^23^. Its principle is simple and easy to use, but it is not applicable to a case where sensitivity analysis is performed on a set of inputs^24–27^.

The composition of SAR SoS in threatening environments is complex, and the SoS capability is affected by many factors. Therefore, in this paper, after analyzing the characteristic of this type of SAR SoS, according to the indicator decomposition hierarchy model, proposes an SPS sensitivity analysis method for the influence degree evaluation.

As a data-based analysis method, the SPS sensitivity analysis method can perform sensitivity analysis on multiple factors simultaneously, the range of factor variations can be extended to the whole defined domain interval, and the range of variation of each factor can be different and multiple factors can change simultaneously, thus it owns a larger model input space and better comparability of analysis results.

Moreover, the advantage of this method is not limited by the model structure, and it does not require an in-depth understanding of the intrinsic mechanism of the model, thus it is more suitable for SoSID analysis of high-dimensional, nonlinear, nonmonotonic models and complex systems, and can be carried out by analytical or simulation methods.

**Figure 2:**
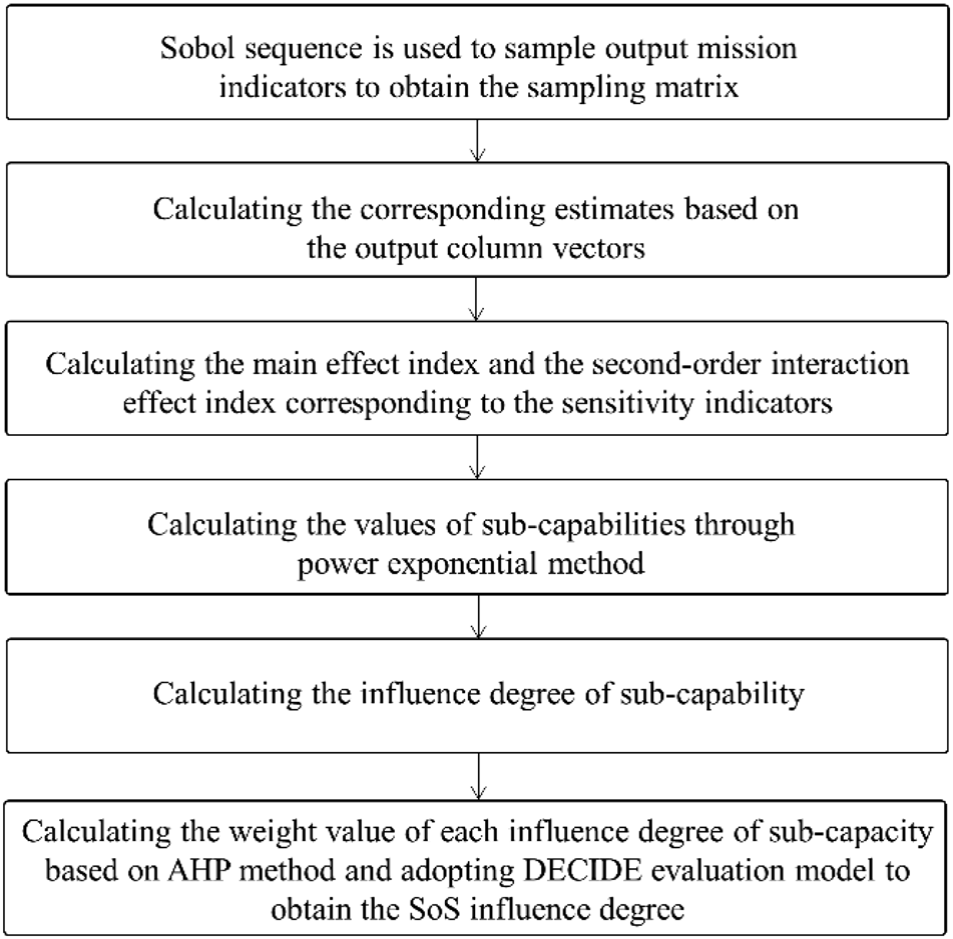
Flow diagram of the SPS analysis method.

The SPS analysis method is based on the idea of model decomposition, which can obtain the first order and higher orders sensitivity of parameters. Its core idea is variance partitioning, which decomposes the model into individual parameter and functions of the combinations between parameters, and analyzes the importance of parameters and the interaction effects between parameters by calculating the influence of the variance of individual input parameter or input parameter sets on the total output variance. The key steps are shown in Fig. 2.

### Calculation process of the SPS analysis method

The detailed processes are shown as follows:

**Step 1** Sample the output mission indicators *N* times using the Sobol sequence to obtain the *N* × 2*n* dimension sampling matrix, where n is the number of mission indicators, and *N* is the number of mission indicators sampled. The first n columns of the matrix are set as matrix *A*, and the last n columns are set as matrix *B*, as shown in Eq. (5). Thus matrix *A* and matrix *B* are both *N* × *n* dimension matrix.

5 $$ A = \left[ {\begin{array}{*{20}c} {x_{11} } & {x_{{1{2}}} } & \cdots & {x_{1n} } \\ {x_{21} } & {x_{22} } & \cdots & {x_{2n} } \\ \vdots & \vdots & \ddots & \vdots \\ {x_{N1} } & {x_{N2} } & \cdots & {x_{Nn} } \\ \end{array} } \right],\quad B = \left[ {\begin{array}{*{20}c} {x^{\prime}_{11} } & {x^{\prime}_{{1{2}}} } & \cdots & {x^{\prime}_{1n} } \\ {x^{\prime}_{21} } & {x^{\prime}_{22} } & \cdots & {x^{\prime}_{2n} } \\ \vdots & \vdots & \ddots & \vdots \\ {x^{\prime}_{N1} } & {x^{\prime}_{N2} } & \cdots & {x^{\prime}_{Nn} } \\ \end{array} } \right] $$

where $x_{ij}$ and $x^{\prime}_{ij}$ are the *i*th sampled values of the *j*th mission indicator within matrix *A* and matrix *B*, respectively.

**Step 2** Calculate the corresponding estimates based on the output column vectors.

Assume that the model is $Y = f\left( X \right)$, where $X = \left[ {x_{1} ,x_{2} , \ldots ,x_{i} , \ldots ,x_{n} } \right]$, $i = 1,2, \ldots n$, and $x_{i}$ is the mission indicator vector. If the square of $f\left( X \right)$ is integrable, the model can be decomposed as follows:

6 $$ \begin{aligned} f\left( X \right) & = f_{0} + \sum\limits_{i = 1}^{n} {\sum\limits_{{r_{1} < \cdots < r_{i} }}^{N} {f_{{r_{1} ,r_{2} , \ldots ,r_{i} }} \left( {x_{{r_{1} }} ,x_{{r_{2} }} , \ldots ,x_{{r_{i} }} } \right)} } \\ & = f_{0} + \sum\limits_{{r_{i} }} {f_{{r_{i} }} } \left( {x_{{r_{i} }} } \right) + \sum\limits_{{r_{i} < r_{j} }} {f_{{r_{i} ,r_{j} }} \left( {x_{{r_{i} }} ,x_{{r_{j} }} } \right)} + \cdots + f_{{r_{1} ,r_{2} , \ldots ,r_{n} }} \left( {x_{{r_{1} }} ,x_{{r_{2} }} , \ldots ,x_{{r_{n} }} } \right) \\ \end{aligned} $$

The decomposition of the model is unique and called variance partitioning if Eq. (6) satisfies $\int_{0}^{1} {f_{{r_{1} ,r_{2} , \ldots ,r_{s} }} \left( {x_{{r_{1} }} ,x_{{r_{2} }} , \ldots ,x_{{r_{s} }} } \right)} dx_{p} = 0$, where $1 \le s \le n$ and $r_{1} \le p \le r_{s}$.

The *i*th column of matrix *B* is replaced by the *i*th column of matrix *A*, the resulting matrix is denoted as $M_{i}$, and the *i*th column of matrix *A* is replaced by the *i*th column of matrix *B*, and the resulting matrix is denoted as $M_{{{ - }i}}$, as shown in Eq. (7), also $M_{i,j}$ and $M_{{{ - }i, - j}}$ can be defined in the same way. The column vectors of the output values are denoted as $Y_{A}$, $Y_{B}$ and $Y_{M}$, corresponding to the input matrix values.

7 $$ M_{i} = \left[ {\begin{array}{*{20}c} {x^{\prime}_{11} } & {x^{\prime}_{12} } & \cdots & {\begin{array}{*{20}c} {x_{1i} } & \cdots & {x^{\prime}_{1n} } \\ \end{array} } \\ {x^{\prime}_{21} } & {x^{\prime}_{22} } & \cdots & {\begin{array}{*{20}c} {x_{2i} } & \cdots & {x^{\prime}_{2n} } \\ \end{array} } \\ \vdots & \vdots & \vdots & {\begin{array}{*{20}c} \vdots & \ddots & \vdots \\ \end{array} } \\ {x^{\prime}_{N1} } & {x^{\prime}_{N2} } & \cdots & {\begin{array}{*{20}c} {x_{Ni} } & \cdots & {x^{\prime}_{Nn} } \\ \end{array} } \\ \end{array} } \right],\quad M_{ - i} = \left[ {\begin{array}{*{20}c} {x_{11} } & {x_{12} } & \cdots & {\begin{array}{*{20}c} {x^{\prime}_{1i} } & \cdots & {x_{1n} } \\ \end{array} } \\ {x_{21} } & {x_{22} } & \cdots & {\begin{array}{*{20}c} {x^{\prime}_{2i} } & \cdots & {x_{2n} } \\ \end{array} } \\ \vdots & \vdots & \vdots & {\begin{array}{*{20}c} \vdots & \ddots & \vdots \\ \end{array} } \\ {x_{N1} } & {x_{N2} } & \cdots & {\begin{array}{*{20}c} {x^{\prime}_{Ni} } & \cdots & {x_{Nn} } \\ \end{array} } \\ \end{array} } \right] $$

The SPS analysis method expresses the effect of all input variables on the model output through the total variance, as shown in Eq. (8).

8 $$ \begin{aligned} V\left( Y \right) & = \frac{1}{N}\sum\limits_{r = 1}^{N} {f^{2} \left( {x_{{r_{1} }} ,x_{{r_{2} }} , \ldots ,x_{{r_{n} }} } \right)} = \frac{1}{N}\sum\limits_{r = 1}^{N} {f\left( {x_{{r_{1} }} ,x_{{r_{2} }} , \ldots ,x_{{r_{n} }} } \right)} \cdot f\left( {x^{\prime}_{{r_{1} }} ,x^{\prime}_{{r_{2} }} , \ldots ,x^{\prime}_{{r_{n} }} } \right) \\ & = \frac{1}{N}Y_{A}^{T} Y_{A} - \frac{1}{N}Y_{A}^{T} Y_{B} = \frac{1}{N}Y_{A}^{T} \left( {Y_{A} - Y_{B} } \right) \\ \end{aligned} $$

Assuming $X_{i}$ to obey a certain distribution, the conditional variance of $Y$ is $V\left( {Y{|}X_{i} } \right)$, which reflects the effect of $X_{i}$ on $Y$, *i.e.* the effect variable $X_{i}$ on the model output. The expect value of the conditional variance $V\left( {Y{|}X_{i} } \right)$ is shown in Eq. (9).

9 $$ \begin{aligned} V\left( {E\left( {Y\left| {X_{i} } \right.} \right)} \right) & = \frac{1}{N}\sum\limits_{r = 1}^{N} {f\left( {x_{{r_{1} }} ,x_{{r_{2} }} , \ldots ,x_{{r_{n} }} } \right)} \cdot f\left( {x^{\prime}_{{r_{1} }} ,x^{\prime}_{{r_{2} }} , \ldots ,x^{\prime}_{{r\left( {i - 1} \right)}} ,x_{ri} ,x^{\prime}_{{r\left( {i + 1} \right)}} , \ldots ,x^{\prime}_{{r_{n} }} } \right) \\ & \quad - \frac{1}{N}\sum\limits_{r = 1}^{N} {f\left( {x_{{r_{1} }} ,x_{{r_{2} }} , \ldots ,x_{{r_{n} }} } \right)} \cdot f\left( {x^{\prime}_{{r_{1} }} ,x^{\prime}_{{r_{2} }} , \ldots ,x^{\prime}_{{r_{n} }} } \right) \\ & = \frac{1}{N}Y_{A}^{T} Y_{{M_{i} }} - \frac{1}{N}Y_{A}^{T} Y_{B} = \frac{1}{N}Y_{A}^{T} \left( {Y_{{M_{i} }} - Y_{B} } \right) \\ \end{aligned} $$

**Step 3** Calculate the main effect index and the second-order interaction effect index corresponding to the sensitivity indicators.

The main effect index and the second-order interaction effect index of the input variables is defined as Eq. (10).

10 $$ \begin{aligned} & S_{{X_{i} }} = \frac{{V\left( {E\left( {Y\left| {X_{i} } \right.} \right)} \right)}}{V\left( Y \right)} \\ & S_{{X_{i} ,X_{j} }} = \frac{{V\left( {E\left( {Y\left| {X_{i} ,X_{j} } \right.} \right)} \right)}}{V\left( Y \right)} = \frac{{V\left( {E\left( {Y\left| {X_{i} X_{j} } \right.} \right)} \right) - V\left( {E\left( {Y\left| {X_{i} } \right.} \right)} \right) - V\left( {E\left( {Y\left| {X_{j} } \right.} \right)} \right)}}{V\left( Y \right)} = \frac{{V\left( {E\left( {Y\left| {X_{i} X_{j} } \right.} \right)} \right)}}{V\left( Y \right)} - S_{{X_{i} }} - S_{{X_{j} }} \\ \end{aligned} $$

where $S_{{X_{{\text{i}}} }}$ is the main effect index of mission indicator $X_{i}$, $S_{{X_{i} X_{j} }}$ is the second-order interaction effect index of $X_{i}$ and $X_{j}$.

**Step 4** Calculate the values of the sub-capabilities through the power exponential method.

After calculating the main effect index values of the evaluation indicators, the power exponential method is used to calculate each sub-capability, as shown in Eq. (11).

11 $$ F(X) = CX_{1}^{{w_{1} }} \cdot X_{2}^{{w_{2} }} \ldots X_{i}^{{w_{i} }} \ldots X_{n}^{{w_{n} }} , $$

where $w_{i}$ is the power index of $X_{i}$, $i = 1,2, \ldots n$; *C* is the correction coefficient, which can be adjusted when comparing the index values between multiple objects or when counting the index values of a collection consisting of different objects, and is generally taken as 1.

When calculating each sub-capability, the main effect value is first used as the power index of the corresponding indicator; *i.e.*, the calculation is performed by substituting $S_{{X_{i} }}$ into $w_{i}$, and the decomposition effect layer indicator $C_{i,j}$ calculation process is obtained as shown in (12).

12 $$ C_{i,j} = X_{1}^{{S_{{X_{1} }} }} \cdot X_{2}^{{S_{{X_{{2}} }} }} \ldots X_{i}^{{S_{{X_{i} }} }} \ldots X_{s}^{{S_{Xs} }} , $$

where $C_{i,j}$ is the *j*th decomposition effect layer indicator within $C_{i}$, *s* is the number of mission indicators within $C_{i,j}$, ${1} \le i \le {6}$, ${1} \le j \le 3$ and ${1} \le s \le {3}$.

Then, the weights of decomposition effect level indicators under the same sub-capability are determined by AHP to give a matrix after a two-by-two comparison, as shown in Eq. (13).

13 $$ P = \left[ {\begin{array}{*{20}c} {a_{11} } & {a_{{1{2}}} } & \cdots & {a_{1n} } \\ {a_{21} } & {a_{22} } & \cdots & {a_{2n} } \\ \vdots & \vdots & \ddots & \vdots \\ {a_{n1} } & {a_{n2} } & \cdots & {a_{nn} } \\ \end{array} } \right], $$

Elements within the matrix *P* satisfy the requirements: $a_{ij} > 0$, $a_{ij} \cdot a_{ji} = 1$, and $a_{ii} = 1$.

The eigenvector of this matrix is calculated, and each value within the eigenvector is used as the power index of each decomposition effect layer indicator. The same method is used to calculate the sub-capability, as shown in Eq. (14).

14 $$ C_{i} = C_{i.1}^{{S_{{C_{i.1} }} }} \cdot C_{i.2}^{{S_{{C_{i.2} }} }} \ldots C_{i.j}^{{S_{{C}_{i.j}} }} \ldots C_{i.t}^{{S_{{C}_{i.t}} }} , $$

where *t* is the number of decomposition effect layer indicators within $C_{i}$, and ${1} \le t \le {3}$.

**Step 5** Calculate the influence degree of sub-capability based on the evaluation model.

The sub-capability influence degree calculation model shown in Eq. (1) is adapted to calculate the change rate of each sub-capability after the input factors change, that is, the influence degree of input factors on the capability of implementing missions, executing actions, destroying enemies, evading enemies, defending against attacks and SAR mission costs.

**Step 6** Calculate the weight of each influence degree of sub-capability based on the AHP method and adopt the DECIDE evaluation model to obtain the whole SoSID.

After the influence degree value of each sub-capability is obtained, the weights are determined by AHP method, the matrix of two-by-two comparison is given, the eigenvector is calculated, the elements within the eigenvector are used as the power index of the influence degree of corresponding sub-capability, and the influence degree of the whole SoS is calculated by the power exponential method and DECIDE evaluation model.

At this point, based on the DECIDE evaluation model and SPS analysis method, the study of the influence degree of relevant input factors on the SAR SoS can be constructed.

## Simulation test and analysis

### Simulation test framework construction

**Figure 3:**
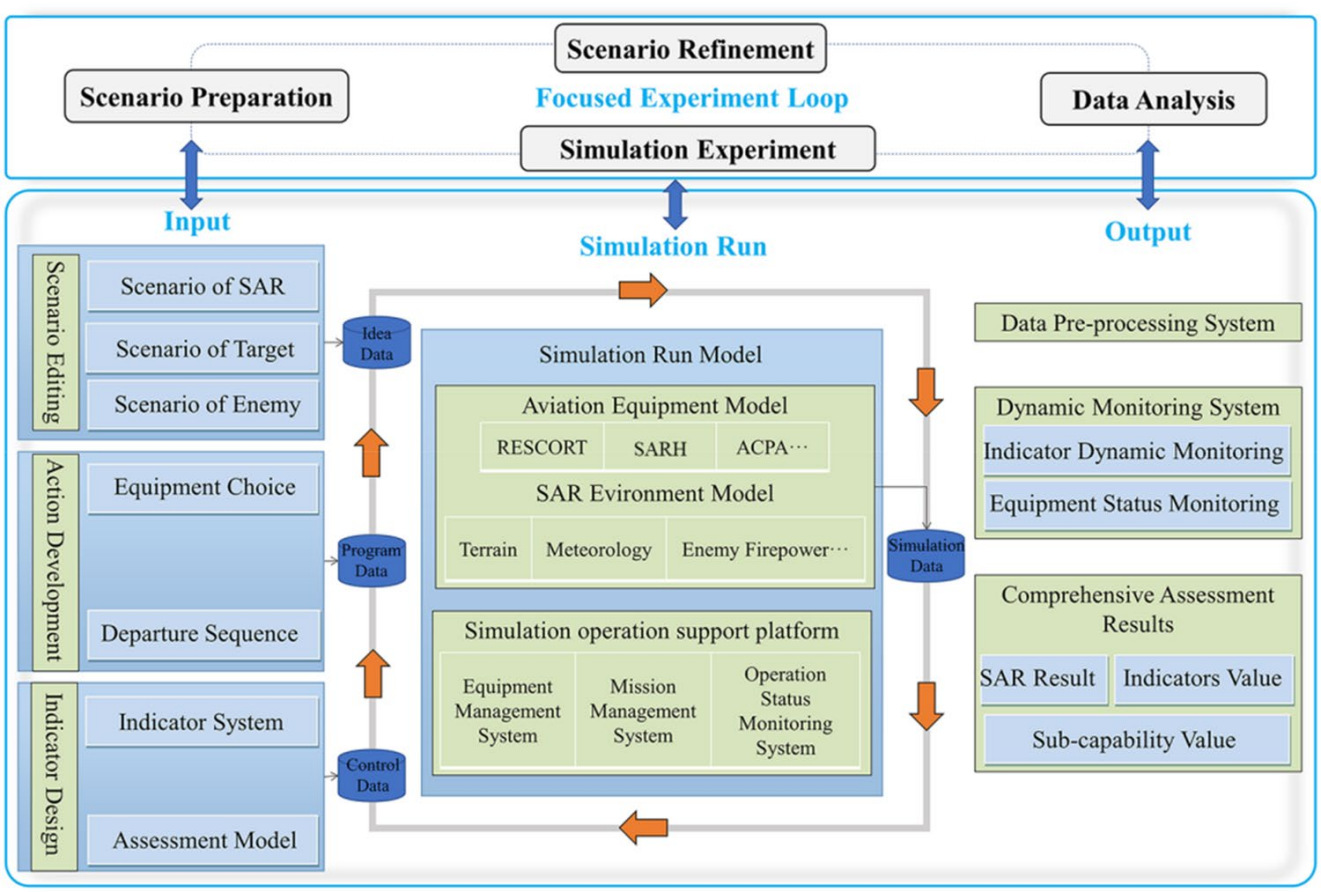
Simulation test environment.

The SAR simulation test framework as shown in Fig. 3, and the aviation equipment or its performance that needs to be verified is added to it (this paper takes the number and performance of aviation equipment as the verification object). The relevant SoS effect are derived through a focused experiment loop with rapid experimental ability to support decision-making.

**Figure 4:**
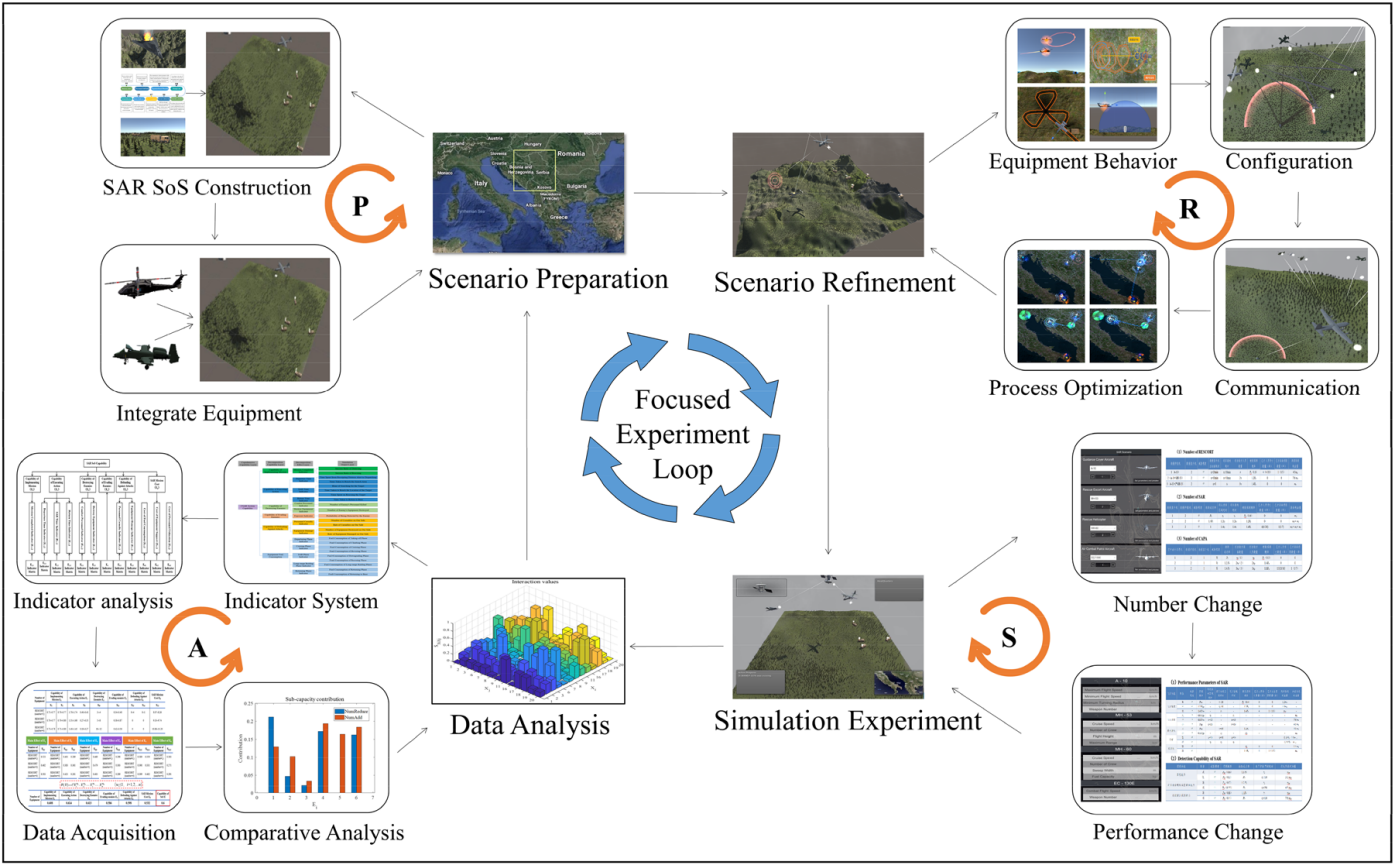
PRSA focused experiment loop.

The focused experiment loop is an entire cycle of the experiment application, consisting of four basic loops: the scenario preparation loop, the scenario refinement loop, the simulation experiment loop, and the data analysis loop (which is defined as PRSA loop in this paper, as shown in Fig. 4).

The focused experiment loop is a gradually approaching the results process from all over the experiment, and researchers can make a judgment based on the goal of the experiment and the results of each experiment cycle and decide whether to conduct the next cycle of the experiment. Through this iteration and gradual approximation, a deeper study for the problem is achieved.

Every test object is gradually added to the SoS, and the SoS test starts from the “existing SoS” and obtains the “difference” compared to the existing SoS. Therefore, it evaluates the whole SoS which is not a local combat operation or a certain equipment system. On this basis, we can analyze and demonstrate how a local change in aviation equipment within SAR forces affects the SoS, which reflects the relationship between the local and the whole, so as to guide the design of aviation equipment and to provide decision support for commanders to conduct SAR force configuration during SAR action.

### Composite modeling based on multiple agents and discrete event system

As a typical complex SoS, SAR SoS has autonomous adaptability, uncertainty and hierarchical emergence. Therefore, it is necessary to build a SAR SoS model based on the characteristics of the SAR SoS and the analysis of the composition of the forces and main actions of the SAR SoS.

Based on the above characteristics, this paper adopts a composite modeling method combining multi-agent and discrete event system (DEVS) modeling to obtain the emergent capability of the SoS through the interaction between agents^28,29^ and realize the temporal logic of the simulation operation through the DEVS, so as to make use of the two complementary functions to obtain a comprehensive consideration of the SAR SoS modeling from two aspects: bottom-up and top-down.

#### Multi-agent modeling

**Figure 5:**
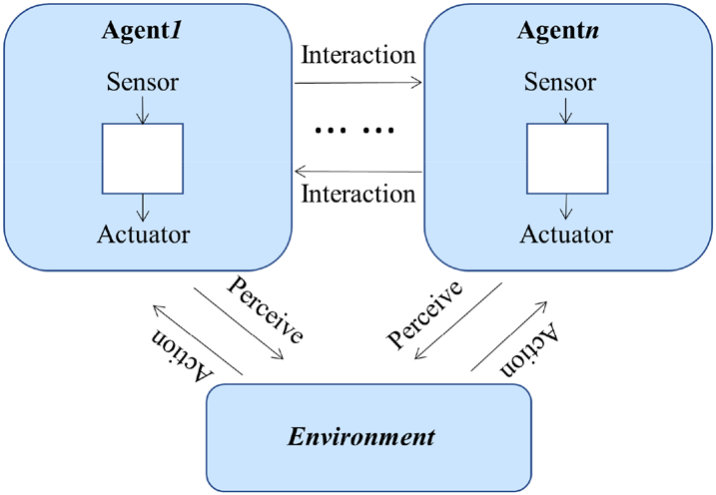
Attributes of an agent.

An agent is an entity that reacts to an object in an environment. It can sense the environment through sensors and influence it through actuators to achieve a complete closed loop, as shown in Fig. 5.

#### DEVS modeling

**Figure 6:**
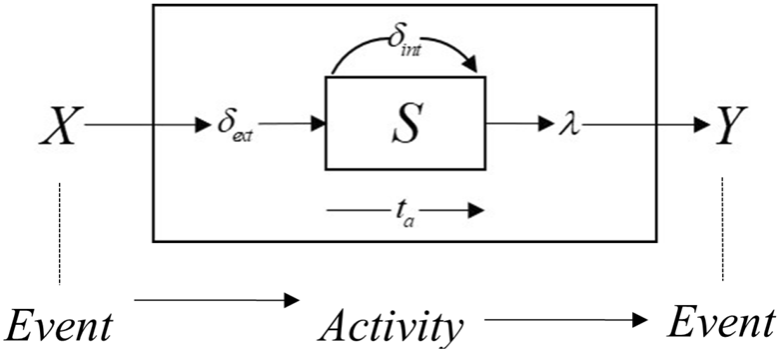
DEVS model.

A SAR operation is very complex and contains not only many independent task phases but also many decision points that need to be evaluated. Therefore, it is difficult to use a purely mathematical approach to analyze the SAR task process. However, the process can be represented as a discrete-event system problem, and the model can be simplified to the form of an “event-activity-event”, as shown in Fig. 6.

In Fig. 6, *X* is the input event set, *Y* is the output event set, *S* is the internal state set, $\delta_{{\text{int}}}$ is the internal state transfer function, $\delta_{ext}$ is the external state transfer function, $\lambda$ is the model output function, and $t_{a}$ is the time advance function.

### Case study

#### Mission scenario construction

**Figure 7:**
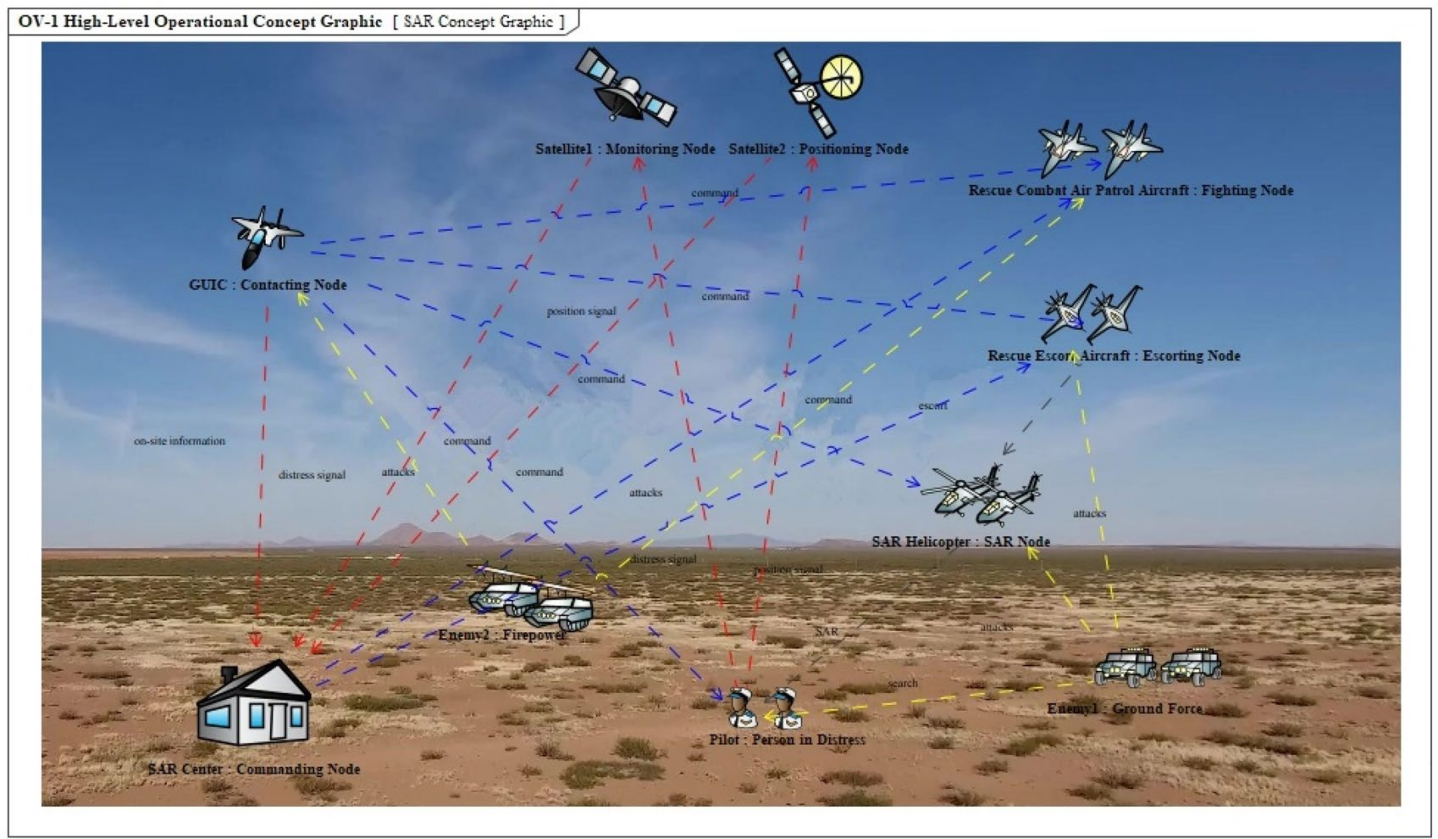
The SAR concept graphic.

This paper evaluates the SAR SoSID of aviation equipment by considering the actions of a warplane being shot down during the war, the pilot falling behind the enemy lines, and then being rescued through the coordinated cooperation of various SAR units, the SAR concept graphic is shown in Fig. 7. The types of aviation equipment in this SAR action include guidance cover aircraft, rescue escort aircraft, search and rescue helicopters, and rescue combat air patrol aircraft^19^. The role and quantity of each piece of the aviation equipment, are shown in Table 2.

**Table 2:** Role and quantity of the aviation equipment.

Equipment type	Abbreviations	Role	Number
Guidance cover aircraft	GUIC	Arrives at the SAR area ahead of search and rescue helicopters to provide guidance and cover	1
Rescue escort aircraft	RESCORT	Provides communication relaying and suppression firepower support for search and rescue helicopters	2
Search and rescue helicopter	SARH	Receives commands from airborne mission command aircraft to rescue personnel in distress	2
Rescue combat air patrol aircraft	RESCAP	Protects SAR crews and personnel in distress from aerial threats	1

#### Scene construction

First, multi-agent modeling is used to construct the entities in the SAR SoS to establish agent models of SAR equipment, targets to be rescued, and enemy firepower, as shown in Table 3.

**Table 3:** Agent model of the aviation equipment, targets and enemy firepower.

Agent	Name	Attribute	Behavior	Perceptual Volume	Meaning
Agent_1_	GUIC	Combat flight speed	Leading and covering	Enemy position	x-Component of enemy position
					z-Component of enemy position
		Weapon number		Target position	x-Component of target position
					z-Component of target position
Agent_2_	RESCORT	Cruise speed	Close air cover	Enemy position	x-Component of enemy position
					z-Component of enemy position
		Maximum flight		SARH position	x-Component of SARH position
					y-Component of SARH position
					z-Component of SARH position
Agent_3_	SARH	Number of crew	Search and rescue	Target position	x-Component of target position
		Fuel capability			
		Sweep width			z-Component of target position
Agent_4_	RESCAP	Combat flight speed	On-site command	Enemy position	y-Component of enemy position
		Weapon number			
Agent_51_ ⋮ Agent_5*n*_	Target_1_ ⋮ Target_*n*_	Remaining life	Evade enemy	Enemy position	x-Component of enemy position
		Evade enemy ability			z-Component of enemy position
		Maximum movement speed	Maintain communication with SAR forces	Command Information	Receive command information
Agent_6_	Enemy Firepower	Position	Attack SAR forces	SAR forces Position	x-Component of SARH position
		Movement speed			y-Component of SARH position
		Movement direction			z-Component of SARH position
		Number of firepower points	Search targets	Target position	Direction of target
		Detection range			Distance of target
		Missile range			Flashing signal Azimuth

Meanwhile, DEVS modeling is used to describe each event during the SAR mission in a discrete manner and to record the changes in the system state that occurred at specific time points. An event-activity-event model is formed as shown in Table 4.

**Table 4:** DEVS model of SAR action.

Event	Description	Activity	Description
*E*_1_	Fighter jet hit by enemy	*A*_1_	Pilot parachutes and sends a distress signal
*E*_2_	Command center receives distress alerts	*A*_2_	GUIC departs to act as field command
*E*_3_	RESCORT receives instructions to depart	*A*_3_	RESCORT departs from base to target airspace
*E*_4_	SARH receives instructions to depart	*A*_4_	SARH departs from base to target airspace
*E*_5_	GUIC observes SARH arriving in target airspace	*A*_5_	Contact distressed personnel to turn on flashing lights
*E*_6_	Personnel in distress receive instructions	*A*_6_	Personnel in distress turn on the flashing lights
*E*_7_	GUIC observes flashing lights	*A*_7_	Command the SARH to carry out rescue
*E*_8_	SARH receives command	*A*_8_	SARH lands and starts rescue
*E*_9_	RESCORT observes SARH beginning descent	*A*_9_	RESCORT advances slowly and takes cover
*E*_10_	Personnel in distress are rescued	*A*_10_	GUIC and RESCORT return to base; SARH sends personnel in distress to the rear hospital

**Figure 8:**
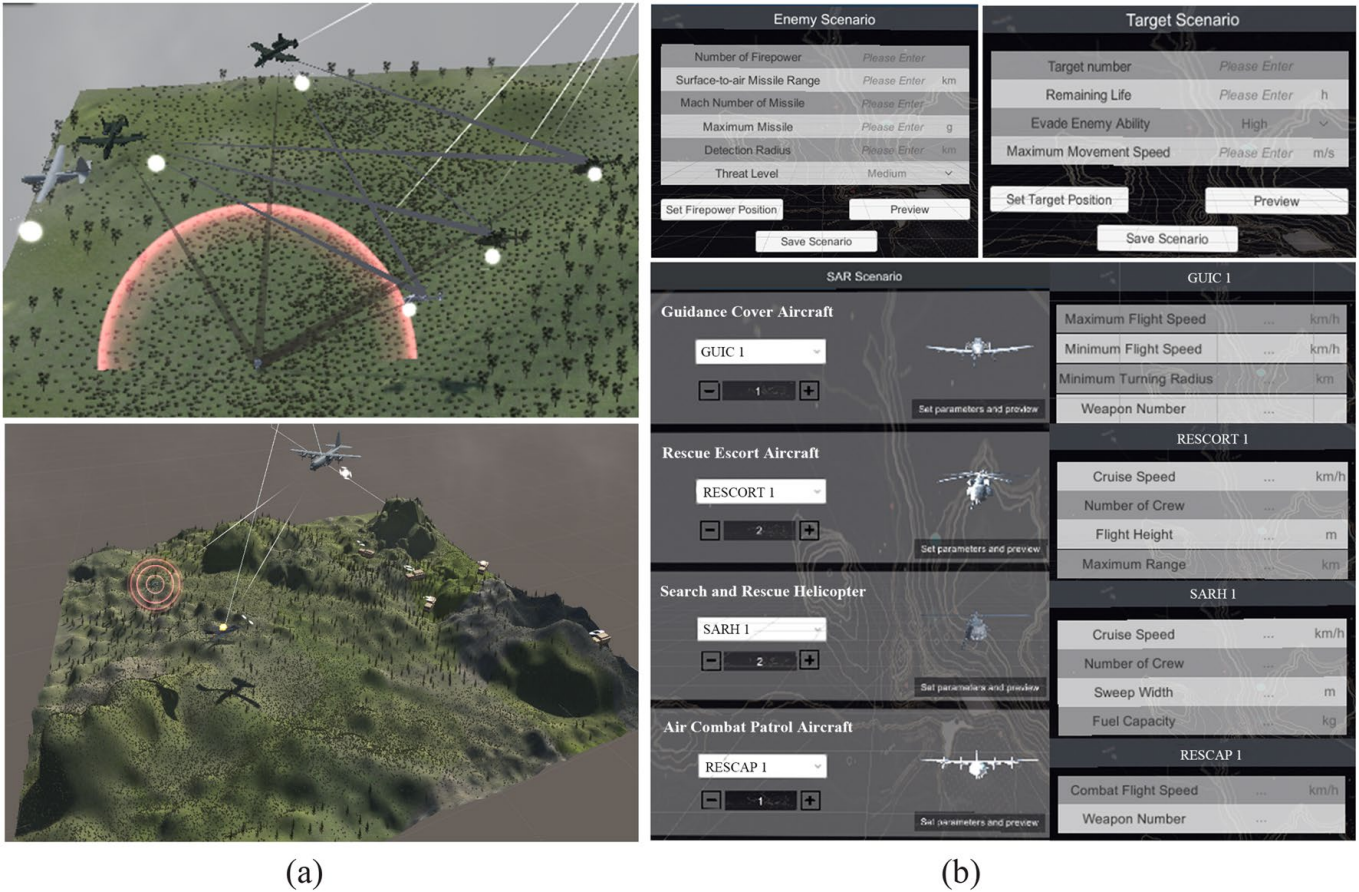
The SAR scenario, scenario editing and parameter setting module.

Based on the simulation test framework and the composite modeling method combining Multi-agent and DEVS, a SAR scenario is constructed as shown in Fig. 8a.

**Figure 9:**
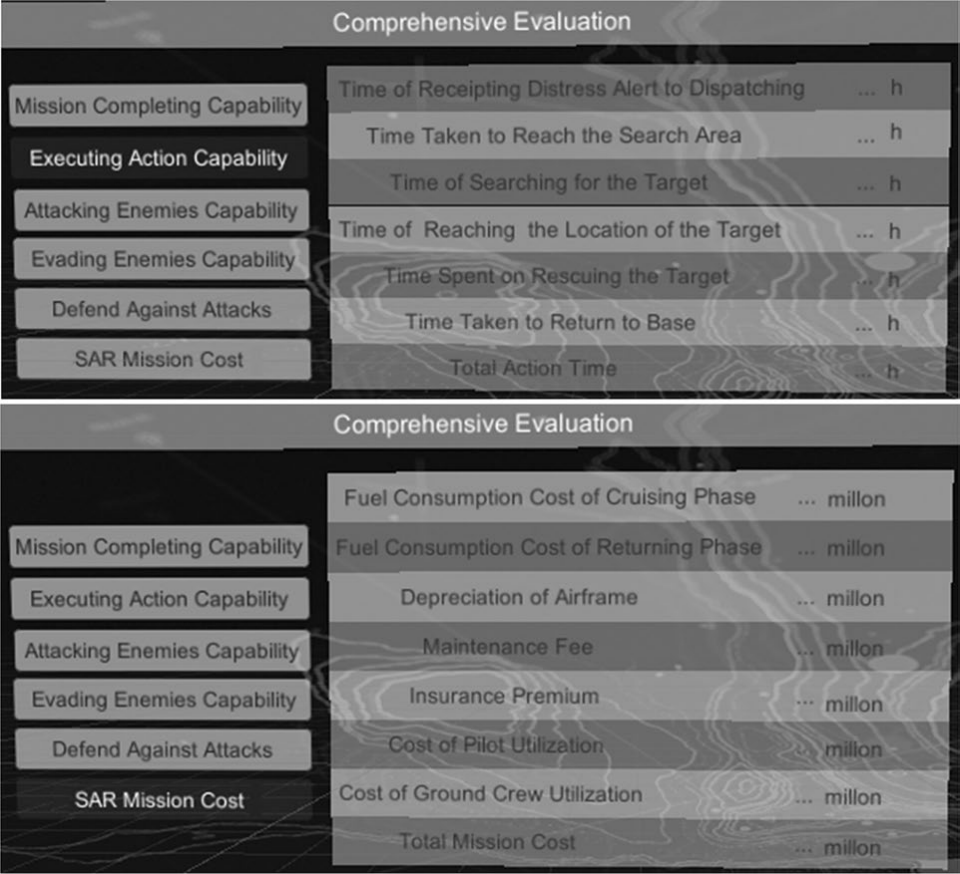
Comprehensive evaluation module.

Moreover, this paper also establishes a scenario editing module (including an enemy scenario, SAR force scenario and target scenario), parameter setting module (including the quantity and performance parameter of each kind of aviation equipment), as shown in Fig. 8b, and comprehensive evaluation module, as shown in Fig. 9.

#### Processing and analysis of simulation results

**Figure 10:**
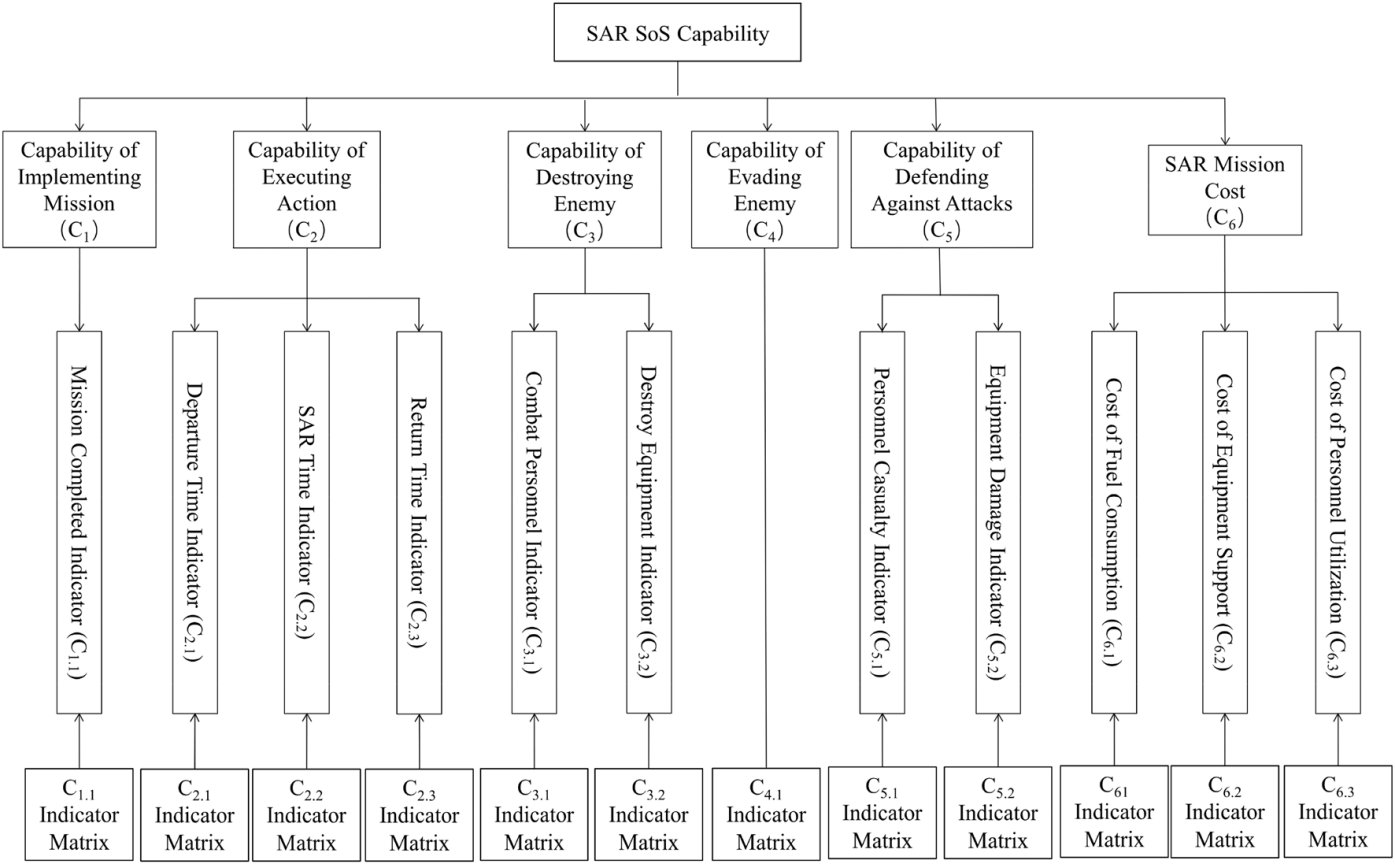
Indicator analysis model.

Before processing the simulation results, the indicator analysis model (as shown in Fig. 10) is refined according to the indicator decomposition hierarchy model shown in Fig. 1, keeping the affiliation relationships unchanged to complete the mapping conversion from the indicator decomposition hierarchy model to the evaluation analysis model.

Specifically, the SAR SoS capability $C_{SoS}$ is decomposed into six sub-capabilities: $C_{1}$, $C_{2}$, $C_{{3}}$, $C_{{4}}$, $C_{{5}}$, $C_{{6}}$. Then the sub-capability is further decomposed to obtain the decomposition effect layer indicator $C_{i,j}$ and its indicator matrix, which is composed of the corresponding mission indicator $X_{i}$.

Based on the refined indicator analysis model, each layer of capability is analyzed in the subsequent analysis process according to the SPS analysis method to evaluate the influence degree of the aviation equipment on the SAR SoS.

**Figure 11:**
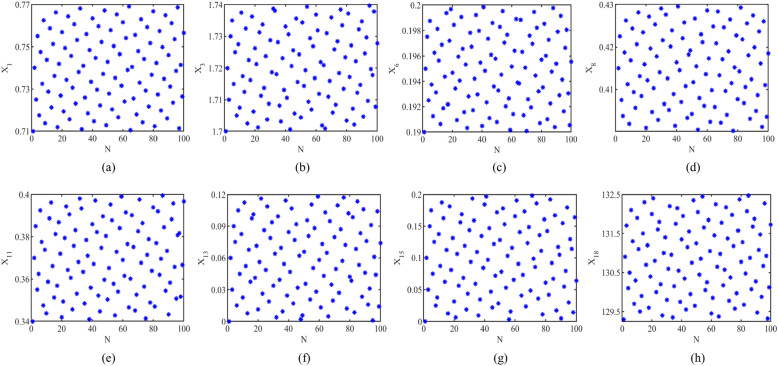
Sampling by the Sobol sequence.

First, the range of the variation interval of each indicator $X_{i}$ in the indicator matrix in this experiment is determined, and the variation range of the values of nineteen indicators are shown in Tables 5 and 6. Then, $X_{i}$ is sampled within its variation interval, and in this paper, $X_{i}$ is sampled *N* (*N* = 100) times by the sobol sequence. Some of the sampling results are shown in Fig. 11. This replaces the pseudorandom sequence in the traditional Monte Carlo method by a quasirandom sequence, overcoming the drawbacks such as the existence of “gaps” within the sample points that reduce its uniformity in distribution brought by pseudorandom sequence sampling. While the quasirandom sequence in the sobol sequence sampling is more uniform in generating random values than the pseudorandom sequence, which results in higher convergence speed and accuracy than the traditional method.

**Table 5:** Indicators $X_{{1}} {{\sim }}X_{{{13}}}$ and their value ranges.

Number	Mission indicator									
	$C_{1}$		$C_{{2}}$				$C_{{3}}$	$C_{{4}}$	$C_{{5}}$	
	$X_{1}$	$X_{2}$	$X_{3} /h$	$X_{{6}} /h$	$X_{{7}} /h$	$X_{{8}} /h$	$X_{{9}}$	$X_{{{1}1}}$	$X_{{1{2}}}$	$X_{{1{3}}}$
**RESCORT**										
2	0.71–0.77	0.70–0.77	1.70–1.74	0.19–0.20	0.19–0.20	0.40–0.43	3–4	0.34–0.40	0–4	0–0.12
3	0.70–0.77	0.70–0.80	1.51–1.60	0.22–0.24	0.19–0.20	0.27–0.35	5–8	0.38–0.87	0–1	0–0.03
4	0.73–0.78	0.71–0.80	1.40–1.45	0.26–0.27	0.19–0.20	0.19–0.27	10–12	0.42–0.56	0–2	0–0.05
**SARH**										
1	0.71–0.77	0.51–0.59	1.51–1.60	0.22–0.24	0.19–0.20	0.27–0.35	5–8	0.37–0.46	0–1	0–0.03
2	0.70–0.77	0.73–0.79	1.51–1.60	0.22–0.24	0.19–0.20	0.33–0.41	6–8	0.43–0.57	0–2	0–0.06
3	0.69–0.77	0.90–1.00	1.52–1.59	0.22–0.23	0.19–0.20	0.39–0.46	5–8	0.51–0.66	0–4	0–0.10

$X_{1}$-success ratio of detection, $X_{2}$-success ratio of rescue, $X_{3}$-time spent from receiving the distress alert to dispatching, $X_{{6}}$-time taken to reach the location of the target to be rescued, $X_{{7}}$-time spent on rescuing the target, $X_{{8}}$-time taken to return to base, $X_{{9}}$-number of enemy personnel killed, $X_{{{11}}}$-probability of being detected by the enemy, $X_{{{12}}}$-number of casualties on our side, $X_{{{13}}}$-rate of casualties on our side.

**Table 6:** Indicators $X_{{{14}}} {{\sim }}X_{{{22}}}$ and their value ranges.

Number	Mission indicator								
	$C_{{5}}$		$C_{{6}}$ (10^–2^ million)						
	$X_{{1{4}}}$	$X_{{1{5}}}$	$X_{{1{6}}}$	$X_{{1{7}}}$	$X_{{1{8}}}$	$X_{{1{9}}}$	$X_{{{20}}}$	$X_{{{21}}}$	$X_{{{22}}}$
**RESCORT**									
2	0–1	0–0.20	10.7–10.9	0.79–0.88	129.3–132.5	33.2–34.0	53.9–55.3	18.4–18.8	8.07–8.26
3	0–1	0–0.17	11.9–12.2	0.76–1.03	196.0–204.9	49.2–51.4	81.7–85.4	21.0–21.9	9.33–9.74
4	0–1	0–0.14	13.4–13.6	0.91–1.19	269.1–277.3	66.8–68.8	112.2–115.6	24.2–24.9	10.9–11.2
**SARH**									
1	0–1	0–0.20	11.5–11.8	0.67–0.91	191.5–199.3	47.6–49.5	79.8–83.1	17.9–18.7	7.86–8.17
2	0–1	0–0.17	12.0–12.3	0.78–1.04	197.2–205.1	49.5–51.5	82.2–85.5	21.2–22.1	9.43–9.80
3	0–1	0–0.14	12.4–12.7	0.92–1.19	202.8–210.5	51.3–53.3	84.6–87.8	24.2–25.2	10.9–11.3

$X_{{{14}}}$-number of pieces of equipment destroyed on our side, $X_{{{15}}}$-rate of equipment damaged on our side, $X_{{{16}}}$-fuel consumption cost of cruising phase, $X_{{{17}}}$-fuel consumption cost of returning phase, $X_{{{18}}}$-depreciation of airframe, $X_{{{19}}}$-maintenance fee, $X_{{{20}}}$-insurance premium, $X_{{{21}}}$-cost of pilot utilization, $X_{{{22}}}$-cost of ground crew utilization. In addition, the indicators with small variation intervals or take essentially constant values would not be listed in this paper.

Next, the main effect value $S_{{X_{i} }}$ of indicator $X_{i}$ is calculated according to the first-order effect index formula (10). The main effect index values are shown in Tables 7 and 8.

**Table 7:** Main effect value of indicators $X_{{1}} {{\sim }}X_{{{12}}}$.

Number	Main effect								
	$S_{{X_{{1}} }}$	$S_{{{\text{X}}_{{2}} }}$	$S_{{{\text{X}}_{{3}} }}$	$S_{{{\text{X}}_{{6}} }}$	$S_{{{\text{X}}_{{7}} }}$	$S_{{{\text{X}}_{{8}} }}$	$S_{{{\text{X}}_{{9}} }}$	$S_{{{\text{X}}_{{{11}}} }}$	$S_{{{\text{X}}_{{{12}}} }}$
**RESCORT**									
2	0.4322	0.5740	0.0511	0.2332	0.2386	0.4652	1.0116	1.0202	0.4303
3	0.3460	0.6573	0.0425	0.0932	0.0327	0.8258	1.0073	1.0044	0
4	0.2441	0.7580	0.0101	0.011	0.0207	0.9540	1.0180	1.0116	0
**SARH**									
1	0.2439	0.7578	0.0425	0.0932	0.0327	0.8258	1.0073	1.0152	0
2	0.6070	0.4024	0.0565	0.1238	0.0435	0.7698	1.0116	1.0118	0
3	0.5310	0.4755	0.0613	0.0571	0.0777	0.7966	1.0073	1.0129	0.4303

**Table 8:** Main effect value of indicators $X_{{{13}}} {{\sim }}X_{{{22}}}$.

Number	Main effect									
	$S_{{{\text{X}}_{{{13}}} }}$	$S_{{{\text{X}}_{{{14}}} }}$	$S_{{{\text{X}}_{{{15}}} }}$	$S_{{{\text{X}}_{{{16}}} }}$	$S_{{{\text{X}}_{{{17}}} }}$	$S_{{{\text{X}}_{{{18}}} }}$	$S_{{{\text{X}}_{{{19}}} }}$	$S_{{{\text{X}}_{{{20}}} }}$	$S_{{{\text{X}}_{{{21}}} }}$	$S_{{{\text{X}}_{{{22}}} }}$
**RESCORT**										
2	0.4281	0.4303	0.4281	0.0293	0.9657	0.3636	0.3006	0.3325	0.5688	0.4614
3	0	0	0	0.0069	0.9906	0.3503	0.3162	0.3319	0.5473	0.4692
4	0	0	0	0.0030	0.9940	0.3578	0.3090	0.3305	0.5326	0.4895
**SARH**										
1	0	0	0	0.0069	0.9907	0.3516	0.3133	0.3332	0.5523	0.4660
2	0	0	0	0.0062	0.9911	0.3524	0.3124	0.3334	0.5582	0.4603
3	0.4281	0.4303	0.4281	0.0063	0.9908	0.3542	0.3100	0.3339	0.5333	0.4849

After calculating the main effect value $S_{{X_{i} }}$ corresponding to the mission indicator $X_{i}$ in each indicator matrix, the power exponential method is used to calculate each decomposition effect layer indicator (such as the mission completed indicator $C_{{{1}{\text{.1}}}}$, departure time indicator $C_{{{2}{\text{.1}}}}$, ……, and cost of personnel utilization $C_{{{6}{\text{.3}}}}$), and the results are obtained as shown in Table 9.

**Table 9:** Value of decomposition effect level indicators $C_{{{1}{\text{.1}}}} {{\sim }}C_{{{6}{\text{.3}}}}$.

Number	$C_{{\text{i,j}}}$											
	$C_{{{1}{\text{.1}}}}$	$C_{{{2}{\text{.1}}}}$	$C_{{{2}{\text{.2}}}}$	$C_{{{2}{\text{.3}}}}$	$C_{{{3}{\text{.1}}}}$	$C_{{{3}{\text{.2}}}}$	$C_{{{4}{\text{.1}}}}$	$C_{{{5}{\text{.1}}}}$	$C_{{{5}{\text{.2}}}}$	$C_{{{6}{\text{.1}}}}$	$C_{{{6}{\text{.2}}}}$	$C_{{{6}{\text{.3}}}}$
**RESCORT**												
2	0.735	1.028	0.461	0.662	3.449	1	0.357	0.223	0.179	0.901	63.97	13.90
3	0.744	1.019	0.826	0.379	6.794	1	0.513	0	0	0.912	96.00	15.42
4	0.758	1.004	0.953	0.245	11.38	1	0.479	0	0	1.058	131.0	17.82
**SARH**												
1	0.588	1.019	0.826	0.377	6.487	1	0.408	0	0	0.805	93.43	13.13
2	0.746	1.026	0.775	0.452	7.263	1	0.495	0	0	0.925	96.63	15.76
3	0.842	1.028	0.809	0.508	6.079	1	0.593	0.252	0.206	1.071	99.77	17.75

Then, the weight of each decomposition effect layer indicator is calculated, and the two-by-two comparison matrix $P_{{\text{i}}}$ is given to calculate the maximum eigenvalue $\lambda_{{\text{i}}}$ and the eigenvector $W_{i}$, as shown in Eqs. (15)–(18).

15 $$ P_{{2}} = \left[ {\begin{array}{*{20}c} {1} & {{{1} \mathord{\left/ {\vphantom {{1} {3}}} \right. \kern-\nulldelimiterspace} {3}}} & {{{1} \mathord{\left/ {\vphantom {{1} {2}}} \right. \kern-\nulldelimiterspace} {2}}} \\ {3} & {1} & {{{3} \mathord{\left/ {\vphantom {{3} {2}}} \right. \kern-\nulldelimiterspace} {2}}} \\ {2} & {{{2} \mathord{\left/ {\vphantom {{2} {3}}} \right. \kern-\nulldelimiterspace} {3}}} & {1} \\ \end{array} } \right],\quad \lambda_{{2}} = {3}{\text{.0,}}\quad W_{2} = \left[ {\begin{array}{*{20}c} {0.2673} & {0.8018} & {0.5345} \\ \end{array} } \right] $$

16 $$ P_{{3}} = \left[ {\begin{array}{*{20}c} {1} & {{{1} \mathord{\left/ {\vphantom {{1} {5}}} \right. \kern-\nulldelimiterspace} {5}}} \\ {5} & {1} \\ \end{array} } \right],\quad \lambda_{{3}} = {2}{\text{.0,}}\quad W_{3} = \left[ {\begin{array}{*{20}c} {0.{1961}} & {0.{9806}} \\ \end{array} } \right] $$

17 $$ P_{{5}} = \left[ {\begin{array}{*{20}c} {1} & {3} \\ {{{1} \mathord{\left/ {\vphantom {{1} {3}}} \right. \kern-\nulldelimiterspace} {3}}} & {1} \\ \end{array} } \right],\quad \lambda_{{5}} = {2}{\text{.0,}}\quad W_{{5}} = \left[ {\begin{array}{*{20}c} {0.9{487}} & {0.{3162}} \\ \end{array} } \right] $$

18 $$ P_{{6}} = \left[ {\begin{array}{*{20}c} {1} & {{{5} \mathord{\left/ {\vphantom {{5} {3}}} \right. \kern-\nulldelimiterspace} {3}}} & {5} \\ {{{3} \mathord{\left/ {\vphantom {{3} {5}}} \right. \kern-\nulldelimiterspace} {5}}} & {1} & {{{3} \mathord{\left/ {\vphantom {{3} {2}}} \right. \kern-\nulldelimiterspace} {2}}} \\ {{{1} \mathord{\left/ {\vphantom {{1} {5}}} \right. \kern-\nulldelimiterspace} {5}}} & {{{2} \mathord{\left/ {\vphantom {{2} {3}}} \right. \kern-\nulldelimiterspace} {3}}} & {1} \\ \end{array} } \right],\quad \lambda_{{6}} = {3}{\text{.05,}}\quad W_{{6}} = \left[ {\begin{array}{*{20}c} {{0}{\text{.8804}}} & {{0}{\text{.4192}}} & {{0}{\text{.2218}}} \\ \end{array} } \right] $$

where $P_{{2}}$, $P_{{3}}$, $P_{{5}}$, $P_{{6}}$ are the two-by-two comparison matrices of decomposition effect layer indicators within $C_{{2}}$, $C_{{3}}$, $C_{{5}}$, $C_{{6}}$, respectively.

**Figure 12:**
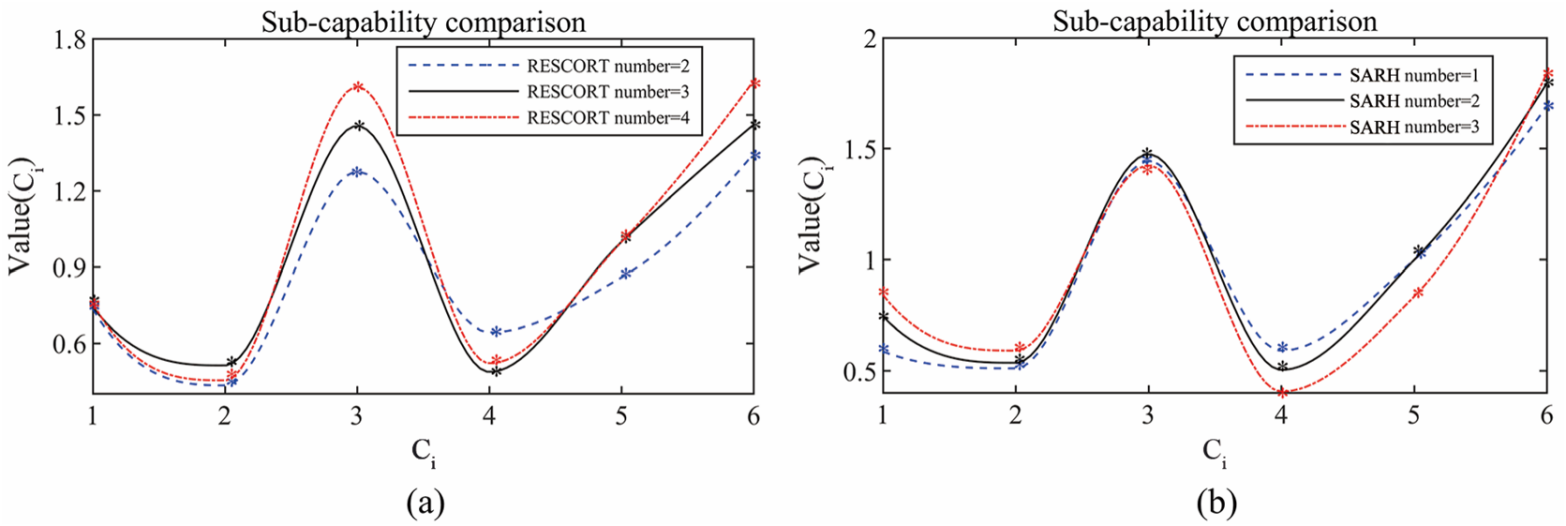
Comparison of sub-capabilities with different numbers of RESCORT or SARH: (**a**) RESCORT, (**b**) SARH.

By taking the elements of each eigenvector as the power index of the corresponding indicators, the sub-capabilities ($C_{{1}}$, $C_{{2}}$, $C_{{3}}$, $C_{{4}}$, $C_{{5}}$, $C_{{6}}$) are calculated by the power index method, and the calculation results are shown in Table 10 and Fig. 12.

**Table 10:** Value of sub-capabilities $C_{{1}} {{\sim }}C_{{6}}$ with different equipment numbers.

Number	$C_{{\text{i}}}$					
	$C_{{1}}$	$C_{{2}}$	$C_{{3}}$	$C_{{4}}$	$C_{{5}}$	$C_{{6}}$
**RESCORT**						
2	0.735	0.434	1.275	0.643	0.860	1.348
3	0.744	0.513	1.456	0.487	1	1.462
4	0.758	0.454	1.611	0.521	1	1.636
**SARH**						
1	0.588	0.512	1.443	0.592	1	1.694
2	0.746	0.537	1.475	0.505	1	1.798
3	0.842	0.592	1.425	0.407	0.836	1.845

Figure 12a shows that after changing the number of RESCORT, there is little change in the capability of implementing missions and the capability of executing actions. The capability of destroying enemies increases linearly with the number of RESCORT increases, that is because that a larger number of RESCORT can cover a larger operational area during the escort process; thus, more enemy deployments can be detected and timely strikes can be executed. Meanwhile, increasing the number of RESCORT can eliminate more threats for SARH and personnel to be rescued because of advance observation and timely strikes of RESCORT against enemy forces, which reduces the probability of SAR forces and targets being detected by the enemy. In addition, the loss of personnel and equipment is reduced while the capability of defending against attacks is increased. However, with continued additional RESCORT (e.g., increasing the value from three to four), the capability of evading enemies and the capability of defending against attacks do not improve significantly; instead, it will increase the cost of SAR missions to a greater extent.

Figure 12b shows that after changing the number of SARH, there is little change in the capability of destroying enemies and the SAR mission costs. In addition, the capability of implementing missions and the capability of executing actions increase with an increase in the number of SARH, which is because when the number of SARH increases, multiple SARH can collaborate in the SAR process.. The SARH can even undertake escort missions, thus improving the efficiency and success rate of a SAR operation, therefore, the capability of implementing missions and the capability of executing actions are improved. In addition, when the number of SARH is increased, the capability of defending against attacks is reduced, which is because there is an increased likelihood of personnel damage and equipment destruction, thus making the SAR force less able to resist enemy attacks.

• Capability of evading enemies corresponds to the probability of not being detected by enemies. Thus, $C_{{4}}$ and $C_{{{4}{\text{.1}}}}$ are opposite. The same calculation process is used for $C_{{5}}$.

**Figure 13:**
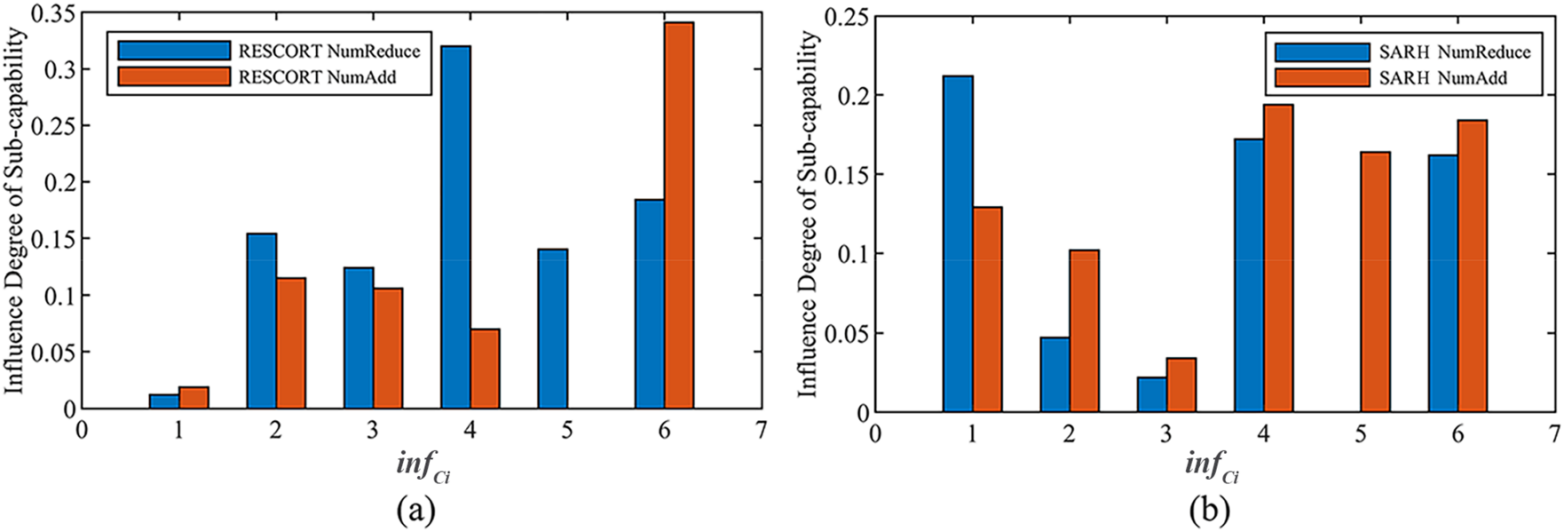
Extent to which the reduction and increase in the number of RESCORT or SARH affects each sub-capability: (**a**) RESCORT, (**b**) SARH.

Furthermore, after obtaining the value of each sub-capability, the rate of change (i.e., influence degree) of each sub-capability when changing input factors was calculated by the influence degree of the sub-capability calculation model shown in Eq. (1), and the calculation results are shown in Table 11 and Fig. 13.

**Table 11:** Influence degree value of each sub-capability when changing the number of equipment.

Number change	$\inf_{{C_{i} }}$						
	$\inf_{{C_{1} }}$	$\inf_{{C_{2} }}$	$\inf_{{C_{3} }}$	$\inf_{{C_{4} }}$	$\inf_{{C_{5} }}$	$\inf_{{C_{6} }}$	$\inf_{SoS}$
RESCORT.Num Reduce	0.012	0.154	0.124	0.320	0.140	0.184	0.159
RESCORT.Num Add	0.019	0.115	0.106	0.070	0	0.341	0.143
SARH.Num Reduce	0.212	0.047	0.022	0.172	0	0.162	0.174
SARH.Num Add	0.129	0.102	0.034	0.194	0.164	0.184	0.214

Table 11 and Fig. 13a show that compared to increasing the number of RESCORT, decreasing the number of RESCORT has a greater influence on each sub-capability, which is because after decreasing the number of RESCORT (e.g., decreasing the value from three to two), the SARH sometimes needs to play the role (such as performing observation, evading and attacking against enemy forces actions during the rescue process as well as during the long-range raid) of RESCORT during the SAR mission, ,which inevitably affects the execution of the main tasks, i.e., SAR, and thus the value of each sub-capability will decrease, especially the capability of evading enemies. The reason why increasing the number of RESCORT (e.g., increasing the value from three to four) has less influence on each sub-capability is that before increasing the number of RESCORT, other aircraft such as RESCAP and GUIC can play the role of RESCORT at the necessary moment to execute the corresponding tasks, which ensures the normal execution of SAR action of SARH; thus, each sub-capability value is less affected.

As seen from Table 11 and Fig. 13b, compared to reducing the number of SARH, increasing the number of SARH has a greater influence on each sub-capability, as the synergy of multiple SARH in the process of performing SAR missions after increasing the number of SARH (e.g., increasing the value from two to three) leads to a greater change in the overall SAR capabilities (especially the capability of executing action, capability of attacking enemies, and capability of defending against attacks).

In summary, the best equipment combination for this SAR case is one GUIC, three RESCORT, two SARH and one RESCAP.

Finally, according to the DECIDE evaluation model, the influence degree of the number of RESCORT and the number of SARH on the overall SAR SoS is calculated. The two-by-two comparison matrix *P*, the maximum eigenvalue $\lambda$ and the eigenvector *W* for each sub-capability are shown in Eqs. (19) and (20).

19 $$ P = \left[ {\begin{array}{*{20}c} 1 & 1 & 7 & 7 & 7 & {{7 \mathord{\left/ {\vphantom {7 3}} \right. \kern-\nulldelimiterspace} 3}} \\ 1 & 1 & 5 & 5 & 5 & {{5 \mathord{\left/ {\vphantom {5 3}} \right. \kern-\nulldelimiterspace} 3}} \\ {{1 \mathord{\left/ {\vphantom {1 7}} \right. \kern-\nulldelimiterspace} 7}} & {{1 \mathord{\left/ {\vphantom {1 5}} \right. \kern-\nulldelimiterspace} 5}} & 1 & {{1 \mathord{\left/ {\vphantom {1 3}} \right. \kern-\nulldelimiterspace} 3}} & {{1 \mathord{\left/ {\vphantom {1 2}} \right. \kern-\nulldelimiterspace} 2}} & {{1 \mathord{\left/ {\vphantom {1 2}} \right. \kern-\nulldelimiterspace} 2}} \\ {{1 \mathord{\left/ {\vphantom {1 7}} \right. \kern-\nulldelimiterspace} 7}} & {{1 \mathord{\left/ {\vphantom {1 5}} \right. \kern-\nulldelimiterspace} 5}} & 3 & 1 & 1 & 3 \\ {{1 \mathord{\left/ {\vphantom {1 7}} \right. \kern-\nulldelimiterspace} 7}} & {{1 \mathord{\left/ {\vphantom {1 5}} \right. \kern-\nulldelimiterspace} 5}} & 2 & 1 & 1 & 3 \\ {{3 \mathord{\left/ {\vphantom {3 7}} \right. \kern-\nulldelimiterspace} 7}} & {{3 \mathord{\left/ {\vphantom {3 5}} \right. \kern-\nulldelimiterspace} 5}} & 2 & {{1 \mathord{\left/ {\vphantom {1 3}} \right. \kern-\nulldelimiterspace} 3}} & {{1 \mathord{\left/ {\vphantom {1 3}} \right. \kern-\nulldelimiterspace} 3}} & 1 \\ \end{array} } \right], $$

20 $$ \lambda = {6}{\text{.6,}}\quad W = \left[ {\begin{array}{*{20}c} {{0}{\text{.7622}}} & {{0}{\text{.5567}}} & {{0}{\text{.0821}}} & {{0}{\text{.2108}}} & {{0}{\text{.1872}}} & {{0}{\text{.1511}}} \\ \end{array} } \right] $$

The DECIDE evaluation model generates an SoSID of 0.159 for the decrease in the number of RESCORT to the overall SAR SoS, 0.143 for the increase in the number of RESCORT to the overall SAR SoS, 0.174 for the decrease in the number of SARH to the overall SAR SoS, and 0.214 for the increase in the number of SARH to the overall SAR SoS. This is in line with the trend of the above influence degree of sub-capability.

**Figure 14:**
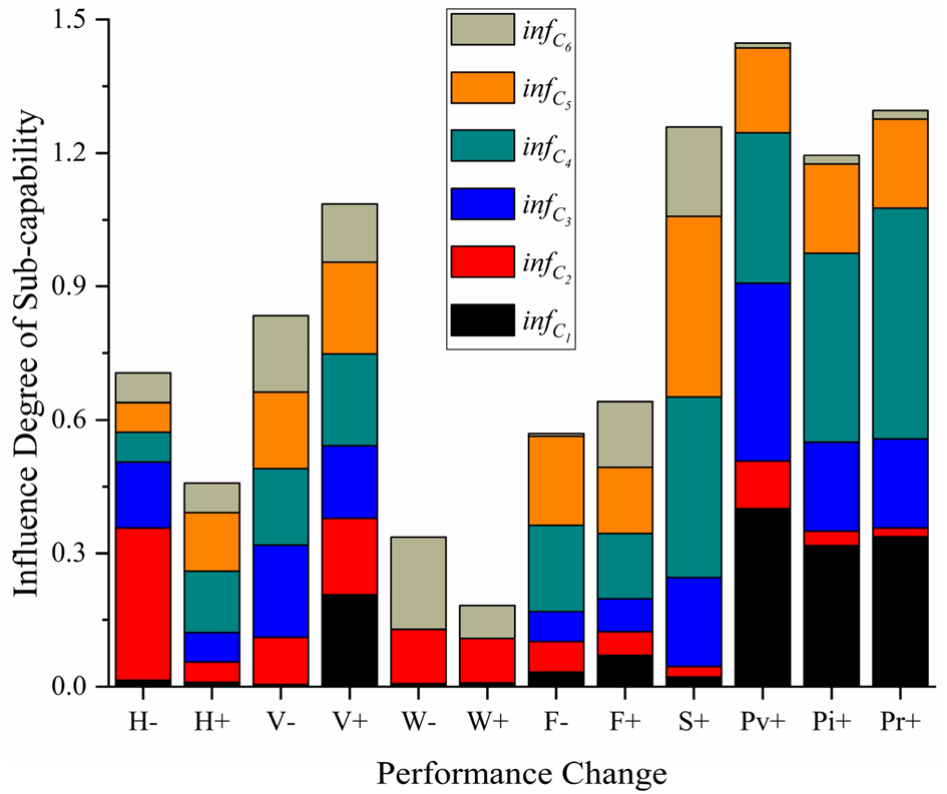
Influence degree of each sub-capability when changing the performance parameters of SARH.

The same method is used to explore the SAR capability values and the influence degree values of each sub-capability and SoS capability under that the SARH owns different performance parameters, and the results are shown in Tables 12 and 13 and Fig. 14.

**Table 12:** Value of sub-capabilities $C_{{1}} {{\sim }}C_{{6}}$ of SARH with different performance parameters.

Performance parameters	$C_{{\text{i}}}$					
	$C_{{1}}$	$C_{{2}}$	$C_{{3}}$	$C_{{4}}$	$C_{{5}}$	$C_{{6}}$
**Cruise height (m)**						
2800	0.710	0.625	1.473	0.570	0.783	1.263
3000	0.721	0.466	1.283	0.608	0.835	1.353
3200	0.728	0.445	1.197	0.692	0.944	1.444
**Cruise speed (km/h)**						
240	0.733	0.372	1.765	0.485	0.685	1.670
290	0.737	0.416	1.462	0.586	0.827	1.425
350	0.890	0.484	1.211	0.707	0.998	1.720
**Takeoff weight (kg)**						
4819	0.745	0.459	1.611	0.611	0.737	1.422
5178	0.740	0.409	1.613	0.610	0.739	1.634
5953	0.733	0.359	1.616	0.608	0.740	1.755
**Maximum flight (km)**						
600	0.723	0.286	1.252	0.882	0.870	1.002
1010	0.700	0.391	1.452	0.739	0.725	1.687
1159	0.749	0.412	1.667	0.526	0.832	1.937
**Stealthiness**						
High	0.747	0.436	1.712	0.817	0.964	2.069
Low	0.731	0.426	1.427	0.581	0.685	1.724
**Visual detection capability**						
High	0.987	0.444	2.076	0.705	0.981	1.670
Low	0.705	0.401	1.483	0.527	0.824	1.651
**Infrared detection capability**						
High	0.960	0.438	1.854	0.734	0.966	1.872
Low	0.729	0.424	1.545	0.515	0.805	1.835
**Radar detection capability**						
High	0.983	0.457	1.964	0.764	0.976	1.711
Low	0.735	0.448	1.637	0.503	0.813	1.677

**Table 13:** Influence degree value of each sub-capability and entire SoS when changing the performance parameters of SARH.

Number change	$\inf_{{C_{i} }}$						
	$\inf_{{C_{1} }}$	$\inf_{{C_{2} }}$	$\inf_{{C_{3} }}$	$\inf_{{C_{4} }}$	$\inf_{{C_{5} }}$	$\inf_{{C_{6} }}$	$\inf_{SoS}$
Reduce cruise height (H−)	0.015	0.342	0.148	0.067	0.067	0.067	0.112
Increase cruise height (H+)	0.010	0.045	0.067	0.138	0.131	0.067	0.065
Reduce cruise speed (V−)	0.005	0.106	0.207	0.172	0.172	0.172	0.073
Increase cruise speed (V+)	0.207	0.172	0.163	0.206	0.207	0.130	0.108
Lighten takeoff weight (W−)	0.007	0.122	0.001	0.002	0.003	0.207	0.039
Increase takeoff weight (W+)	0.009	0.100	0.002	0.003	0.001	0.074	0.042
Reduce maximum flight (F−)	0.033	0.069	0.067	0.194	0.200	0.006	0.051
Increase maximum flight (F+)	0.070	0.054	0.074	0.147	0.148	0.148	0.044
Improve stealthiness (S+)	0.022	0.023	0.200	0.406	0.407	0.200	0.235
Improve visual detection capability (P_v_+)	0.400	0.107	0.400	0.338	0.191	0.011	0.141
Improve infrared detection capability (Pi+)	0.317	0.033	0.200	0.425	0.200	0.020	0.125
Improve radar detection capability (P_r_+)	0.337	0.020	0.200	0.519	0.200	0.020	0.138

Tables 12 and 13 and Fig. 14 show that the detection capability of the SARH has a greater influence on the capability of implementing missions and the capability of evading enemies. The influence degree values of implementing missions after improving the visual detection capability, infrared detection capability and radar detection capability are 0.400, 0.317 and 0.337, respectively. The corresponding influence degree values of evading enemies are 0.338, 0.425 and 0.519, respectively. Improving the detection capability can significantly improve the detection success rate of personnel and the probability of detecting an enemy threat, thus the capability of implementing missions and the capability of evading enemies are significantly improved. Lowering the cruise altitude, increasing the cruise speed, and reducing the takeoff weight are all conducive to improving the capability of executing action and the capability of destroying enemies, and lowering the cruise altitude is more beneficial than increasing the cruise speed. Lightening the takeoff weight of the SARH is conducive to reducing the overall cost of the SAR mission, so the takeoff weight of the equipment should be reduced as much as possible under the condition of meeting the mission requirements. Improving the stealth of the SARH can improve the ability of the equipment to evade the enemy and defend against resist attacks to a greater extent (the influence degree values reach 0.406 and 0.407, respectively) so that it can SAR more targets under the premise of ensuring its own safety, so the stealth capability of aviation equipment should be improved.

Additionally, from Table 13, it can be seen that reducing the cruise altitude of SARH, increasing their cruise speed, and improving their stealth and detection capability all have a strong influence on the influence degree of the SAR SoS, especially improving the stealth of SARH can improve the capability of the whole SAR SoS to a large extent.

**Figure 15:**
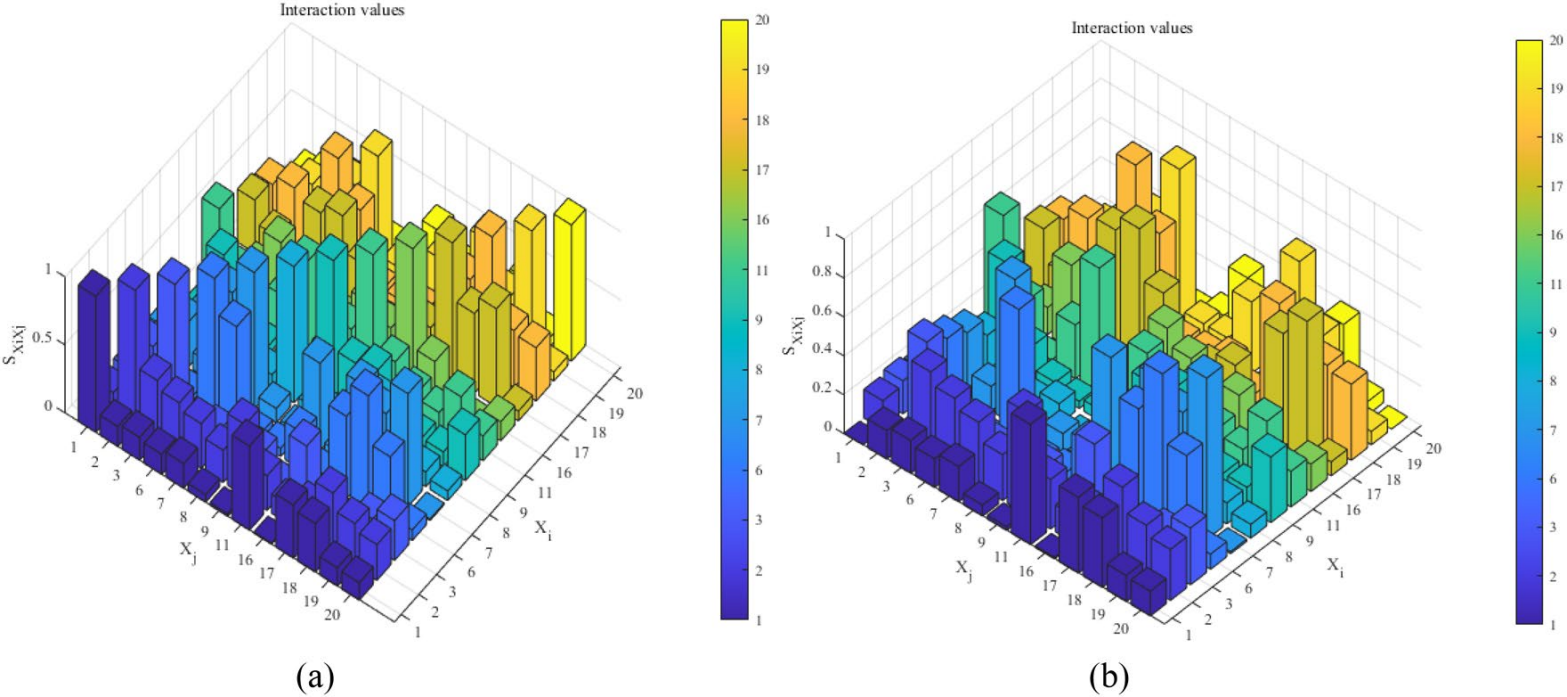
Comparison of the second-order interaction effect indices.

In the actual SAR action, the analysis of the SAR capability focuses not only on individual indicator, but also on the influence degree of the interrelationship between indicators on the SAR capability, usually mainly focuses on the indicators with larger interactions, whereas the relationship between indicators with smaller interactions can often be ignored. Therefore, this paper also calculates and analyzes the second-order interaction effect index in the sensitivity analysis, and the results are shown in Table 14. The values in the table are taken as absolute values to obtain the comparison graph shown in Fig. 15a. The value of the self-interaction effect index (i.e., $S_{{X_{i} X_{i} }}$) is removed to obtain the comparison graph shown in Fig. 15b.

**Table 14:** Second-order interaction effect index value between indicators.

$X_{i}$	$X_{j}$												
	$X_{1}$	$X_{2}$	$X_{{3}}$	$X_{{6}}$	$X_{{7}}$	$X_{{8}}$	$X_{{9}}$	$X_{{{1}1}}$	$X_{{{16}}}$	$X_{{{17}}}$	$X_{{{18}}}$	$X_{{{19}}}$	$X_{{{20}}}$
$X_{1}$	1	0.147	− 0.169	0.148	− 0.182	0.057	− 0.020	0.618	− 0.009	0.390	0.358	0.135	0.128
$X_{2}$		1	0.441	0.379	0.329	0.236	0.527	− 0.255	0.139	0.225	0.435	0.313	− 0.262
$X_{{3}}$			1	− 0.035	0.127	0.091	− 0.170	0.022	− 0.429	0.062	− 0.058	0.201	− 0.292
$X_{{6}}$				1	0.549	0.049	0.061	− 0.276	0.604	0.074	0.517	0.411	0.078
$X_{{7}}$					1	− 0.104	− 0.037	0.641	0.075	0.829	0.415	0.484	0.011
$X_{{8}}$						1	0.057	0.085	0.171	− 0.426	0.097	0.108	0.071
$X_{{9}}$							1	− 0.350	0.399	− 0.095	− 0.106	− 0.108	− 0.337
$X_{{{1}1}}$								1	− 0.316	− 0.189	− 0.149	− 0.367	− 0.183
$X_{{{16}}}$									1	− 0.274	− 0.051	0.012	0.140
$X_{{{17}}}$										1	0.484	0.521	0.072
$X_{{{18}}}$											1	0.579	0.391
$X_{{{19}}}$												1	− 0.068
$X_{{{20}}}$													1

As seen from Fig. 15, the large values of the interaction effect index for the combination of indicators ${{X_{{1}} } \mathord{\left/ {\vphantom {{X_{{1}} } {X_{{{11}}} }}} \right. \kern-\nulldelimiterspace} {X_{{{11}}} }}$, ${{X_{7} } \mathord{\left/ {\vphantom {{X_{7} } {X_{11} }}} \right. \kern-\nulldelimiterspace} {X_{11} }}$, ${{X_{{6}} } \mathord{\left/ {\vphantom {{X_{{6}} } {X_{{1{6}}} }}} \right. \kern-\nulldelimiterspace} {X_{{1{6}}} }}$, ${{X_{{8}} } \mathord{\left/ {\vphantom {{X_{{8}} } {X_{{1{7}}} }}} \right. \kern-\nulldelimiterspace} {X_{{1{7}}} }}$ and ${{X_{{{18}}} } \mathord{\left/ {\vphantom {{X_{{{18}}} } {X_{19} }}} \right. \kern-\nulldelimiterspace} {X_{19} }}$ indicate that the interaction between these indicators has a large influence on SAR capability and thus should be considered comprehensively in the sensitivity analysis process.

## Conclusion

1. This paper first proposes the concept of the “SoSID”, then based on the analysis of the SAR capability, an indicator decomposition hierarchy model and a DECIDE SoSID evaluation model were established, and the SPS analysis method was proposed for the sensitivity analysis of the SAR SoSID of the aviation equipment. Then, the rationality of the established model was verified through a case study, and conclusions with specific guiding significance were drawn.
2. The number of RESCORT affects the execution of SAR action to some extent, especially the capability of destroying enemies, the capability of evading enemies, and the capability of defending against attacks. The number of SARH also affects the execution of SAR action, especially the capability of implementing missions and the capability of executing actions. Moreover, the decrease and increase in the number of RESCORT and SARH have different influence degree on SAR capability, where a decrease in the number of RESCORT causes more changes in overall SAR capability than an increase in the number of RESCORT, whereas an increase in the number of SARH has a greater influence on overall SAR capability than a decrease in the number of SARH. Therefore, when formulating the SAR plans, it needs to consider the scale effect of a certain type of equipment and select a certain amount of equipment for a specific mission to maximize SoS capability.
3. Reducing the cruise altitude of SARH, increasing the cruise speed, and improving their stealth and detection capability are all conducive to improving the capability of the SAR SoS; in particular, improving the stealth of SARH can improve the capability of the whole SAR SoS to a greater extent. Therefore, the designers should improve the stealth of the aviation equipment through various methods during designing process.
4. Adopting the SPS analysis method to compare and analyze the second-order interaction effect index values among indicatorscan determine which interactions among indicators have a greater influence on SAR capability, which would help to pay attention to some important indicators and their relationships, so as to provide corresponding support for SAR force configuration.

## Data Availability

Almost all data generated or analyzed during this study are included in this published article, corresponding author would like to provide more data on reasonable request.
